# Multi-Sensor Geometric Documentation of Cultural Heritage at Risk Across Inland, Coastal and Shallow-Water Environments

**DOI:** 10.3390/s26185698

**Published:** 2026-09-08

**Authors:** Styliani Verykokou, Charalabos Ioannidis, Chryssy Potsiou, Sofia Soile, Konstantinos Tokmakidis, Kimon Papadimitriou, Panagiotis Tokmakidis, Alexandros Tourtas, Salvatore Martino, Guglielmo Grechi, Kyriacos Themistocleous, Sławomir Królewicz, Włodzimierz Rączkowski, Jannis Holzer, Eleonoor Bosch, David Nguyen, Fabien Langenegger, Stefan Plattner, Themistoklis Bilis, Alexander Sokolicek, Markus Gschwind, Doris Lettmann, Agnieszka Oniszczuk

**Affiliations:** 1 Laboratory of Photogrammetry, School of Rural, Surveying and Geoinformatics Engineering, National Technical University of Athens, 15780 Athens, Greece; cioannid@survey.ntua.gr (C.I.); chryssyp@survey.ntua.gr (C.P.); ssoile@survey.ntua.gr (S.S.); 2 Laboratory of Topography (LabTop), School of Rural and Surveying Engineering, Aristotle University of Thessaloniki, 54124 Thessaloniki, Greece; ktok@auth.gr (K.T.); paki@auth.gr (K.P.); ptokmaki@gmail.com (P.T.); alextourtas@gmail.com (A.T.); 3 CERI-Research Center for the Prediction, Prevention, and Control of Geological Risks, Sapienza University of Rome, 00185 Rome, Italy; salvatore.martino@uniroma1.it (S.M.); guglielmo.grechi@uniroma1.it (G.G.); 4 Eratosthenes Centre of Excellence, 3012 Limassol, Cyprus; k.themistocleous@eratosthenes.org.cy; 5 Faculty of Geographical and Geological Sciences, Adam Mickiewicz University in Poznań, 61-680 Poznań, Poland; slawomir.krolewicz@amu.edu.pl; 6 Faculty of Archaeology, Adam Mickiewicz University in Poznań, 61-614 Poznań, Poland; wlodzimierz.raczkowski@amu.edu.pl; 7 Centre Suisse d’Electronique et de Microtechnique SA (CSEM), 2002 Neuchâtel, Switzerland; jannis.holzer@csem.ch (J.H.); eleonoor.bosch@csem.ch (E.B.); david.nguyen@csem.ch (D.N.); 8 OARC, Office de l’Archéologie Cantonale de Neuchâtel, 2068 Hauterive, Switzerland; fabien.langenegger@ne.ch; 9 Remote Sensing Technology Institute (IMF), German Aerospace Center (DLR), 82234 Wessling, Germany; stefan.plattner@dlr.de; 10 German Archaeological Institute (DAI), 10678 Athens, Greece; themistoklis.bilis@dainst.de; 11 Department of Classical Studies, Paris Lodron University of Salzburg, 5020 Salzburg, Austria; alexander.sokolicek@plus.ac.at; 12 Bavarian State Department of Monuments and Sites (BLfD), 80539 Munich, Germany; markus.gschwind@blfd.bayern.de (M.G.); doris.lettmann@blfd.bayern.de (D.L.); 13 National Institute of Cultural Heritage (NID), 00-805 Warsaw, Poland; aoniszczuk@nid.pl

**Keywords:** cultural heritage, multi-sensor documentation, UAV photogrammetry, underwater photogrammetry, flash LiDAR, unmanned surface vehicle, bathymetry, geometric documentation, digital twins, risk-informed conservation

## Abstract

**Highlights:**

**What are the main findings?**

- Multi-sensor geometric documentation produced site-specific 2D, 2.5D and 3D evidence layers across eight inland, coastal and shallow-water cultural heritage sites.
- Optical photogrammetry, acoustic sounding and flash LiDAR provided operationally complementary information on exposed remains, unstable terrain, submerged structures and underwater morphology, addressing different spatial scales, targets and environmental conditions.

**What are the implications of the main findings?**

- Cultural heritage documentation in risk-related contexts should be designed around the conservation question and site conditions, rather than around a single sensor, product type or level of geometric detail.
- The value of documentation products lies in their ability to transform visible, submerged and terrain-related conditions into measurable spatial records for future assessment and monitoring.

**Abstract:**

Climate-related and environmental hazards affect cultural heritage sites in markedly different inland, coastal, lacustrine and underwater settings, creating documentation requirements that cannot be addressed by a single sensing approach. This study presents the multi-sensor geometric documentation of eight cultural heritage sites. Unmanned aerial vehicle (UAV) photogrammetry was applied to six inland and coastal sites, while underwater photogrammetry, unmanned surface vehicles (USVs), acoustic sounding and a prototype green-wavelength flash LiDAR were used at three shallow-water sites. The campaigns produced orthomosaics, elevation models, dense point clouds, textured meshes, bathymetric maps and underwater LiDAR point clouds at scales appropriate to the conservation problem of each site. The resulting products document exposed architectural remains, excavation areas, cliffs and unstable slopes, lake-margin changes, submerged masonry, wooden structures and lakebed morphology. Their main contribution is the establishment of spatially explicit, site-specific baselines that provide measurable geometric and visual evidence for condition assessment, future repeat-survey comparisons and the spatial integration of environmental, archaeological and conservation information. The study demonstrates the operational and information complementarity of optical, acoustic and active ranging approaches, which address different documentation scales, environmental constraints and heritage targets, and provide distinct spatial evidence that can serve as potential inputs to subsequent digital twin and decision support applications.

## 1. Introduction

Cultural heritage sites are increasingly exposed to environmental, climatic, geological and hydrological processes that may affect their preservation, accessibility and long-term management, as highlighted by systematic and recent reviews on climate-change impacts and risk assessment for cultural heritage [1,2,3,4,5]. This is particularly relevant for coastal and low-lying heritage sites, where sea-level rise, flooding and erosion may substantially increase future exposure and management challenges [6,7,8]. These processes do not act uniformly across heritage contexts. Inland archaeological sites may be affected by weathering, material deterioration, soil moisture variations or slope-related phenomena; coastal sites may be exposed to erosion, sea spray, wave action, cliff instability and sea-level-related pressures; shallow-water sites may be affected by sediment dynamics, water-level fluctuations, biological activity and limited accessibility. As a result, the documentation of cultural heritage at risk cannot rely only on descriptive recording or isolated visual inspection. It requires accurate spatial information that captures both the heritage asset and its surrounding environmental context.

Geometric documentation has become a central component of cultural heritage recording, conservation and management. Image-based and scanner-based three-dimensional (3D) modelling technologies can generate detailed representations of objects, monuments, archaeological sites and complex landscapes, supporting metric documentation, visualisation, analysis and dissemination [9,10,11,12,13,14,15,16,17,18]. For large and complex archaeological areas, multi-resolution and multi-sensor documentation strategies have also been shown to be necessary in order to address different scales, levels of detail and recording requirements within the same site [19,20,21]. Recent studies have further emphasized the need for standardized yet adaptable documentation practices, in which the selection of survey methods is related to factors such as site scale and complexity, accessibility, required level of detail and intended use [22,23]. In image-based approaches, the reliability of the final products depends strongly on image acquisition geometry, radiometric quality, image matching, camera orientation and dense reconstruction procedures [24,25,26,27,28,29,30,31,32]. In parallel, the increasing availability of unmanned aerial vehicle (UAV) platforms, terrestrial and mobile mapping sensors, low-cost light detection and ranging (LiDAR) devices and underwater acquisition systems has expanded the range of cultural heritage environments that can be documented in two-dimensional (2D), two-and-a-half-dimensional (2.5D) and 3D representations [33,34,35,36,37,38,39,40].

Recent research has also emphasized that the documentation of cultural heritage should not be limited to visually realistic 3D models. In risk-related contexts, the value of geometric documentation lies in its ability to provide spatial baselines that can be reused for condition assessment, monitoring, interpretation and decision making [41,42,43,44,45,46,47,48,49]. Within the TRIQUETRA research project, this need has been addressed through the integration of environmental, geospatial, archaeological and conservation information for cultural heritage sites exposed to climate-related and natural hazards [50]. Previous work has presented the overall TRIQUETRA approach and has reported the geometric documentation of the Greek pilot sites participating in the project [50,51]. Other TRIQUETRA-related studies have examined coastal heritage resilience through geospatial, land administration, digital twin and decision support perspectives [52], and have demonstrated the use of UAV-derived digital surface models (DSMs) and orthomosaics for flood risk assessment [53]. Further site- and sensor-specific studies have addressed photogrammetric and remote sensing investigations at Ventotene [54,55,56], engineering-geological, geophysical and hazard-modelling investigations at Aegina Kolonna using the geometric documentation results [57,58,59], monitoring and digital representation at Choirokoitia [60], UAV-based environmental monitoring and flood risk modelling using drone-photogrammetric terrain data at Smuszewo [61,62], coastal hazard modelling applications at Epidaurus [59], bathymetric and shallow-water photogrammetric documentation at Roseninsel [63], and underwater flash LiDAR developments and field applications at Epidaurus, Roseninsel and Les Argilliez [64,65]. These studies provide important project-, site- and technology-specific results; however, they do not provide a detailed and consistently structured account of the geometric documentation across all eight pilot sites or an integrated comparative interpretation of the information value and monitoring relevance of the resulting geometric products across heterogeneous heritage settings.

The current state of the art shows important progress in individual sensing technologies and documentation workflows. UAV photogrammetry has been widely used for the documentation of built and archaeological heritage, offering flexible acquisition, high spatial resolution and efficient coverage of complex sites [66,67,68,69,70,71,72,73]. Recent studies have further examined accuracy-controlled UAV documentation, optimized viewpoint planning and UAV photogrammetry–LiDAR comparisons for heritage recording, highlighting the influence of acquisition geometry, control configuration and sensor characteristics on the resulting 3D products [74,75,76]. Underwater photogrammetry has become one of the main non-invasive techniques for submerged cultural heritage documentation, although it remains constrained by visibility, illumination, water depth, radiometric degradation and operational access [31,77,78,79]. Earlier work on underwater archaeological recording has also demonstrated the value of multi-image photogrammetry as an accessible approach for producing metric and visually interpretable 3D records of submerged sites [80,81,82,83,84]. Other approaches, including acoustic remote sensing, LiDAR and multi-sensor fusion, have also been increasingly explored to overcome limitations of purely optical documentation and to extend mapping capabilities in complex or partially inaccessible environments [85,86,87,88,89,90]. In shallow-water cultural heritage, direct comparison of photogrammetry and unmanned surface vehicle (USV)-mounted multibeam echosounding has also shown their complementary information value, with acoustic data providing broader seabed morphology and photogrammetry providing higher-resolution information on archaeological objects and structures [90]. Despite these advances, many studies remain focused on a single site, a single type of environment or a specific sensor configuration, providing limited basis for interpreting how the information value and monitoring relevance of geometric documentation products vary according to the conservation question and environmental setting. Against this background, the relationship between previous TRIQUETRA and related pilot-site publications and the contribution of the present study is made explicit in Table 1. The table summarizes prior coverage and identifies the datasets, methodological reporting, results and study-level integrated interpretation that are newly reported or substantially extended in the present work.

This paper addresses this gap by presenting the multi-sensor geometric documentation of eight cultural heritage pilot sites that differ substantially in setting, scale, material, accessibility and conservation problem. The study includes inland archaeological sites, coastal heritage landscapes and shallow-water cultural heritage environments, including both lacustrine and shallow-marine contexts. UAV photogrammetry was applied to six inland and coastal sites, while underwater photogrammetry, unmanned surface vehicles, acoustic sounding and a prototype green-wavelength flash LiDAR were used at three shallow-water sites. The purpose of the paper is not to propose a universal documentation workflow or to rank the performance of different sensors under controlled benchmark conditions. Likewise, the present study does not perform a new cross-site hazard or risk assessment; rather, it examines how the generated geometric products provide spatial evidence that can be combined with hazard, environmental, material and condition information in subsequent vulnerability and conservation analyses. Within this scope, the study adopts a fit-for-purpose perspective in which site-specific acquisition strategies and the resulting geometric products are interpreted in relation to the conservation question, environmental setting and intended monitoring role. The methodological contribution therefore lies in interpreting the different documentation strategies and resulting geometric products in relation to the type, scale and information value of the spatial evidence they provide across heterogeneous heritage settings.

The scientific innovation of the present study lies in the integrated interpretation of heterogeneous geometric documentation campaigns according to their conservation purpose, rather than in the introduction of a new sensing technology or processing algorithm. First, it provides a consistently structured and substantially more detailed account of the geometric documentation across all eight pilot sites, including datasets and results that are newly reported or substantially extended where applicable, as summarized in Table 1. Second, it interprets orthomosaics, DSMs and digital terrain models (DTMs), dense point clouds, textured meshes, bathymetric datasets and underwater LiDAR point clouds according to their type, scale, information content and relevance to the site-specific conservation question. Third, by considering all eight pilots within the same study, it identifies how documentation requirements and monitoring relevance differ among detailed archaeological recording, documentation of terrain and cliffs, multi-temporal surface monitoring, and documentation of submerged sites and underwater morphology. This integrated comparative interpretation constitutes the principal scientific advance beyond the aggregation of previously reported documentation campaigns.

The remainder of the paper is organized as follows. Section 2 introduces the eight pilot sites, presenting their cultural heritage context, dominant risks and dominant conservation threats. Section 3 describes the data acquisition, processing and product generation activities, including UAV-based photogrammetry, underwater photogrammetry, acoustic sounding and flash LiDAR surveys. Section 4 presents the results, focusing on the generated geometric documentation products, their information value across different site contexts and their relevance as baselines for future monitoring and, where repeated acquisitions are available, as multi-temporal spatial evidence. Section 5 discusses the contribution and limitations of the study, with emphasis on fit-for-purpose documentation, the operational and information complementarity of different sensing approaches, the supporting role of geometric documentation in risk-informed conservation and the potential use of geometric documentation products as spatial inputs to digital twin and decision support environments [91].

## 2. Pilot Sites: Context and Risks

TRIQUETRA’s eight pilot cultural heritage sites span inland monuments, coastal and underwater landscapes and lacustrine settings, covering a wide range of typologies, periods and materials. In this section, we present a concise profile of each site, including its location and cultural heritage context and the principal environmental, geological, hydrological or anthropogenic processes relevant to its conservation.

### 2.1. Kalapodi (Greece)

**Figure 1:**
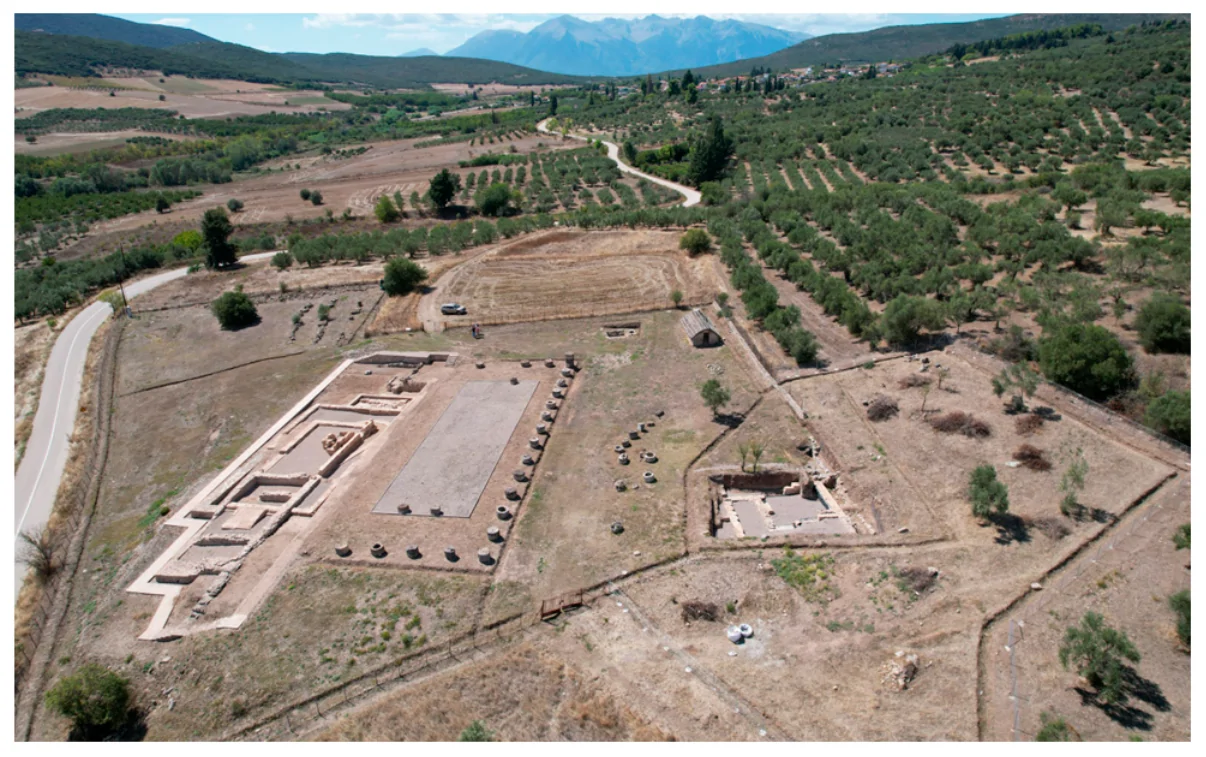
Aerial view of the ancient sanctuary of Kalapodi.

The ancient sanctuary of Kalapodi (Figure 1) is located in central Greece in modern Fthiotida. The archaeological site is situated at an altitude of about 310 m at a distance of about 1300 m east of the modern settlement of Kalapodi. In antiquity, the area belonged to the territory of ancient Phokis. The famous pan-Hellenic oracle of Apollo at Delphi belonged to the same territory. The sanctuary of Kalapodi is most probably another esteemed oracular shrine of Apollo, the one at Abae, one of the most ancient and important sanctuaries of Central Greece. During systematic excavations carried out in the last fifty years under the direction of the German Archaeological Institute, the centre of the sanctuary with two parallel temple complexes and parts of surrounding buildings have been brought to light dating from the Mycenaean period to the Late Roman period (1400 BC to AD 500) [92,93].

The site is remarkable for both the antiquity of the cult and the continuous use of the sacred space over approximately two millennia, represented by more than 13 architectural phases and several provisional sanctuaries. Particularly significant is the existence of a large-scale, architecturally designed space as early as the Mycenaean period. The excavations have also provided important evidence concerning cult practices, animal sacrifices and offerings. The architectural remains of the Early Iron Age are significant for the evolution of ancient Greek architecture. The architecture became gradually more monumental in Geometric and Archaic Times. After the Persian destruction, on the site of the Archaic southern temple, only a small adyton was established, while in the north a large Doric temple was built. In Hellenistic Times, a new building was established northeast of the older temple complex, while the last temple of the Roman period was again built in the south only partially covering the ruin of the old temple complex and allowing access to the Classical adyton. In late antiquity, a settlement had been established on the site [92,93].

The archaeological site at Kalapodi is exposed to various atmospheric and environmental hazards that pose risks to the preservation of its stone structures and materials. Various factors, such as moisture fluctuations and temperature variations, contribute to weathering of the site. Due to its location, in a rural and relatively elevated landscape in the middle of a valley, the Kalapodi sanctuary is also exposed to frost phenomena during several days per year. These freeze–thaw cycles accelerate micro-cracking and scaling in the limestone blocks used in the sanctuary’s buildings, which in turn will gradually weaken the structural integrity. Therefore, long-term conservation is challenging and requires timely mitigation measures [50].

Environmental monitoring and stone-material characterization complement the documentation of the site by providing information on the climatic exposure and material susceptibility of the archaeological remains. These investigations include local meteorological observations and characterization of the limestone properties relevant to weathering and freeze–thaw deterioration [94,95].

### 2.2. Ventotene and Santo Stefano (Italy)

**Figure 2:**
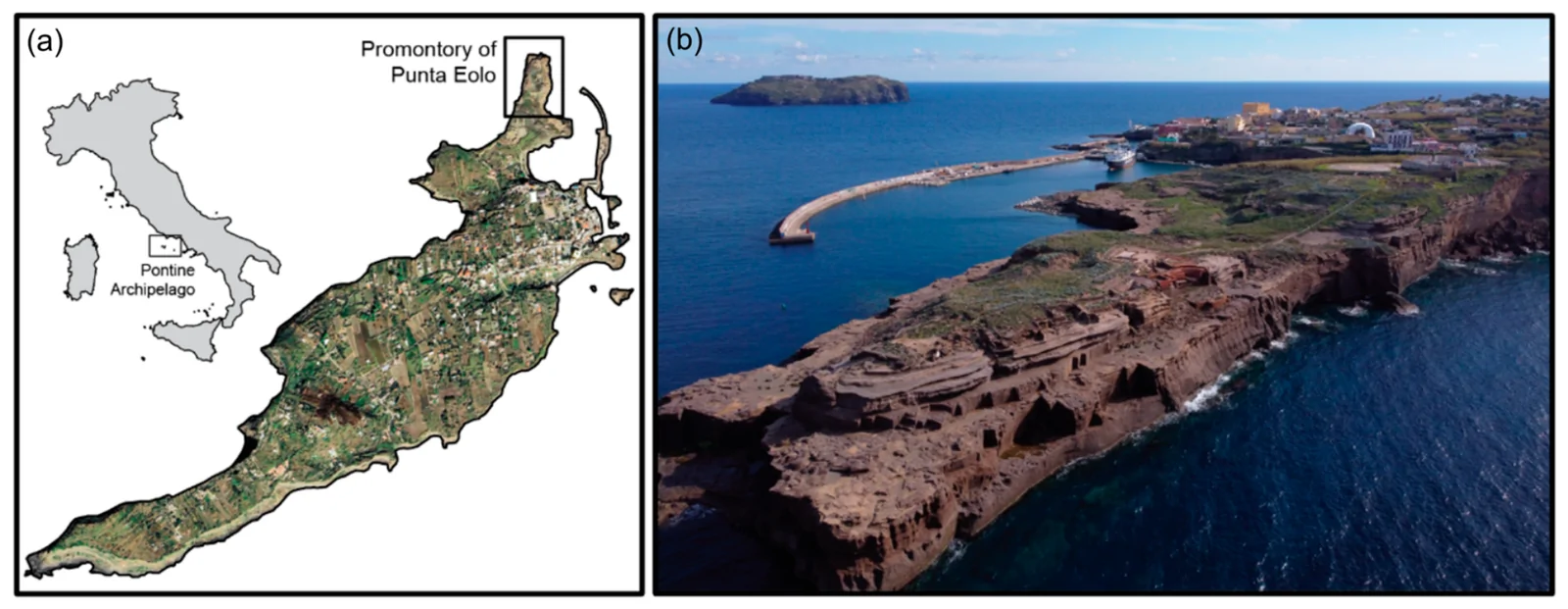
(**a**): Location of Ventotene Island within the Pontine Archipelago, central Italy. (**b**): Unmanned aerial vehicle (UAV)-based oblique view of the Punta Eolo promontory showing the archaeological remains of Villa Giulia and the surrounding plunging coastal cliffs.

The islands of Ventotene and Santo Stefano are part of the Pontine Archipelago (Figure 2a) and represent the emergent portion of a large stratovolcano that once reached an estimated maximum elevation of ~800 m [96]. Ventotene extends for approximately 1.5 km^2^ and features a complex volcanic stratigraphy characterized by alternating lava flows and pyroclastic deposits. While lava units dominate the southwestern coastal sectors, extensive cliffs composed of consolidated pyroclastic deposits occur along the northern and eastern margins of the island, producing pronounced variations in coastal morphology and mechanical properties. Beyond its geological significance, Ventotene holds outstanding historical and cultural value. Since Roman times, the island has served as a place of exile due to its geographic isolation and rugged topography. From the late 1st century BC, several prominent women of the imperial family were banished to the island, most notably Julia the Elder, daughter of Emperor Augustus [54]. These exiles were confined within the Villa Giulia complex, an imperial residence constructed atop the northernmost promontory of the island, Punta Eolo. The site is among the most significant archaeological areas in Ventotene, preserving architectural remains that provide insights into Roman imperial life and coastal settlement strategies. In later centuries, the island continued to serve as a political and penal hub, hosting various detention facilities during the Bourbon and Fascist periods. Today, Ventotene is recognized as a significant cultural and natural heritage site, with the Villa Giulia archaeological complex serving as a key element of its identity (Figure 2b).

The island of Ventotene faces natural hazards closely connected to the dynamics of the coastal environment. Persistent sea wave action and strong wind gusts cause coastal erosion and are key preparatory factors for the initiation of slope instabilities and landslides, inducing coastline retreat and threatening buildings and heritage sites. Besides shaping the shoreline, marine spray and wind-driven salt moisture contribute to the deterioration of archaeological remains, especially at the Villa Giulia complex. Thermal cycling and heat waves can also accelerate material weathering and structural weakening over time. Although wildfires are less common, they pose an additional environmental hazard to the island. In fact, in recent years, several fires have affected areas near the archaeological site [55].

A multidisciplinary investigation of Punta Eolo and the Villa Giulia complex has characterized the geological, geomorphological and archaeological setting of the site [56]. At Punta Eolo in Ventotene, high-resolution UAV-based and terrestrial photogrammetry are combined with geophysical surveys and material characterization analyses to characterize the site’s structural conditions and preservation status. Three-dimensional documentation of the archaeological structures and surrounding coastal cliffs provides the spatial context required for interpreting the site in relation to the surrounding coastal morphology and ongoing hazard analyses. Complementary in situ monitoring provides information on sea-wave forcing and rock-mass conditions relevant to coastal cliff instability [97]. Together, these observations support the interpretation of landslides affecting the coastal cliffs and the development of appropriate conservation strategies.

### 2.3. Aegina (Greece)

The archaeological site of Aegina Kolonna, a prominent cultural heritage landmark in Greece, is located in the northwest of the island of Aegina in the middle of the Saronic Gulf between Attica and the Peloponnesus. Its proximity to the mainland, coupled with its advantageous access to the Aegean Sea, has attracted people to settle since the Neolithic period (at least since the 4th millennium BC). Two natural harbour bays and a fertile hinterland offered a particularly suitable environment for habitation. Consequently, it is home to the largest and most important Bronze Age settlement on the island, dating back to the 3rd and 2nd millennia BC. The earliest remains are concentrated in the centre of the hill. The elaborate architecture and rich finds bear witness to an early complex community that was an active part of the Aegean world through extensive trade networks. In historic times (7th century BC–AD 3rd century) it housed a sanctuary on the hill, separated from the nearby residential area in the south, which was at its peak in Archaic and Early Classical times (6th–5th century BC). A fortified settlement was built in the Byzantine era (AD 6th–10th century) on top of the earlier remains [57].

**Figure 3:**
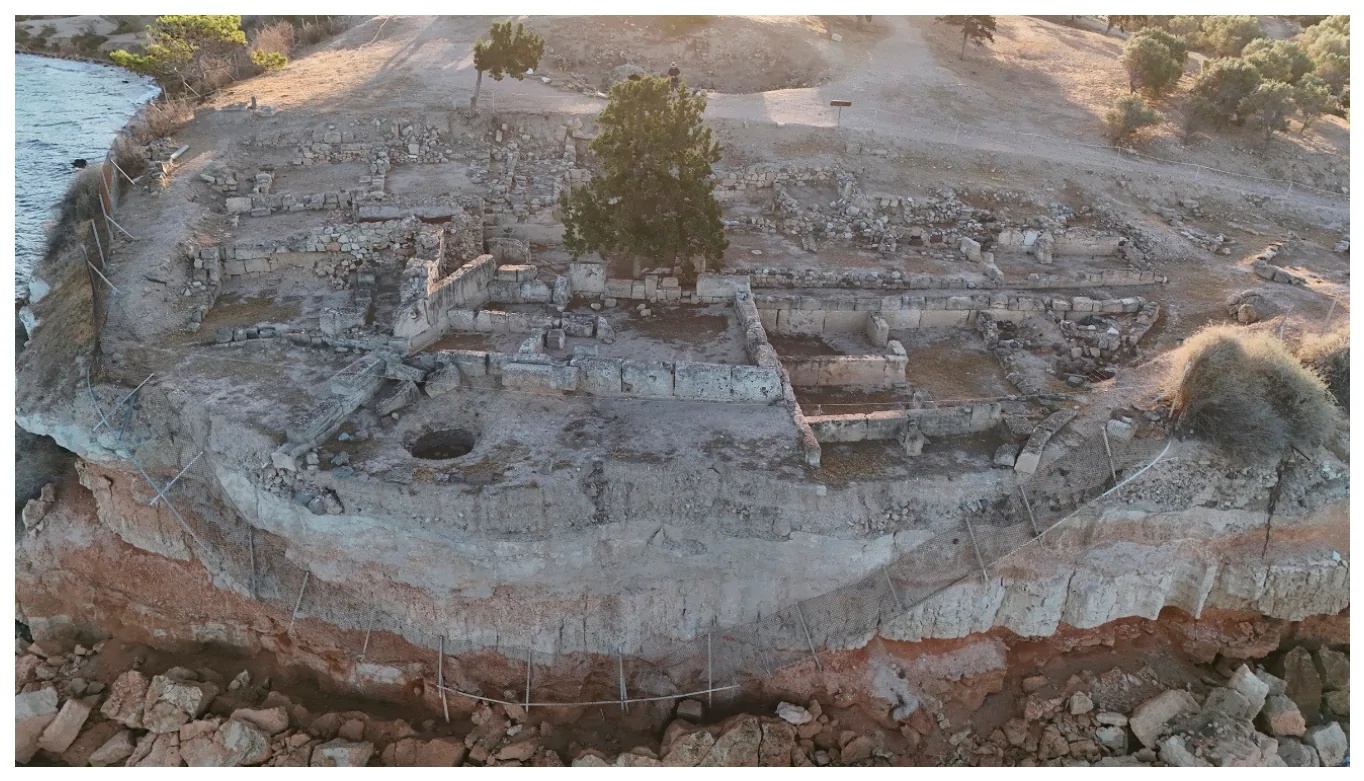
Aegina Kolonna, western part.

The site of Aegina is increasingly threatened by geological hazards, including coastal erosion, seismic activity and slope instabilities (Figure 3). The progressive retreat of the calcarenite sea cliffs has already led to the loss of unexcavated historical remains, posing a severe risk to the site’s long-term preservation. Previous work at Aegina Kolonna combined archaeological investigation with systematic digitization of the excavation, supporting geophysical investigations and site modelling [98,99].

Engineering-geological, geophysical and archaeological investigations have been combined to assess site vulnerability and inform targeted mitigation strategies [57]. A comprehensive geological survey identified the primary factors driving cliff instability, while ambient seismic noise measurements helped characterize the subsurface conditions and preliminarily assess local seismic amplification effects. Conservation concerns also extend to the northeastern prehistoric settlement. Past restoration efforts relied on cement-based mortars, which have deteriorated over time, leading to structural instability. Current conservation measures combine detailed documentation and long-term monitoring with interventions aimed at stabilizing exposed archaeological remains.

### 2.4. Choirokoitia (Cyprus)

**Figure 4:**
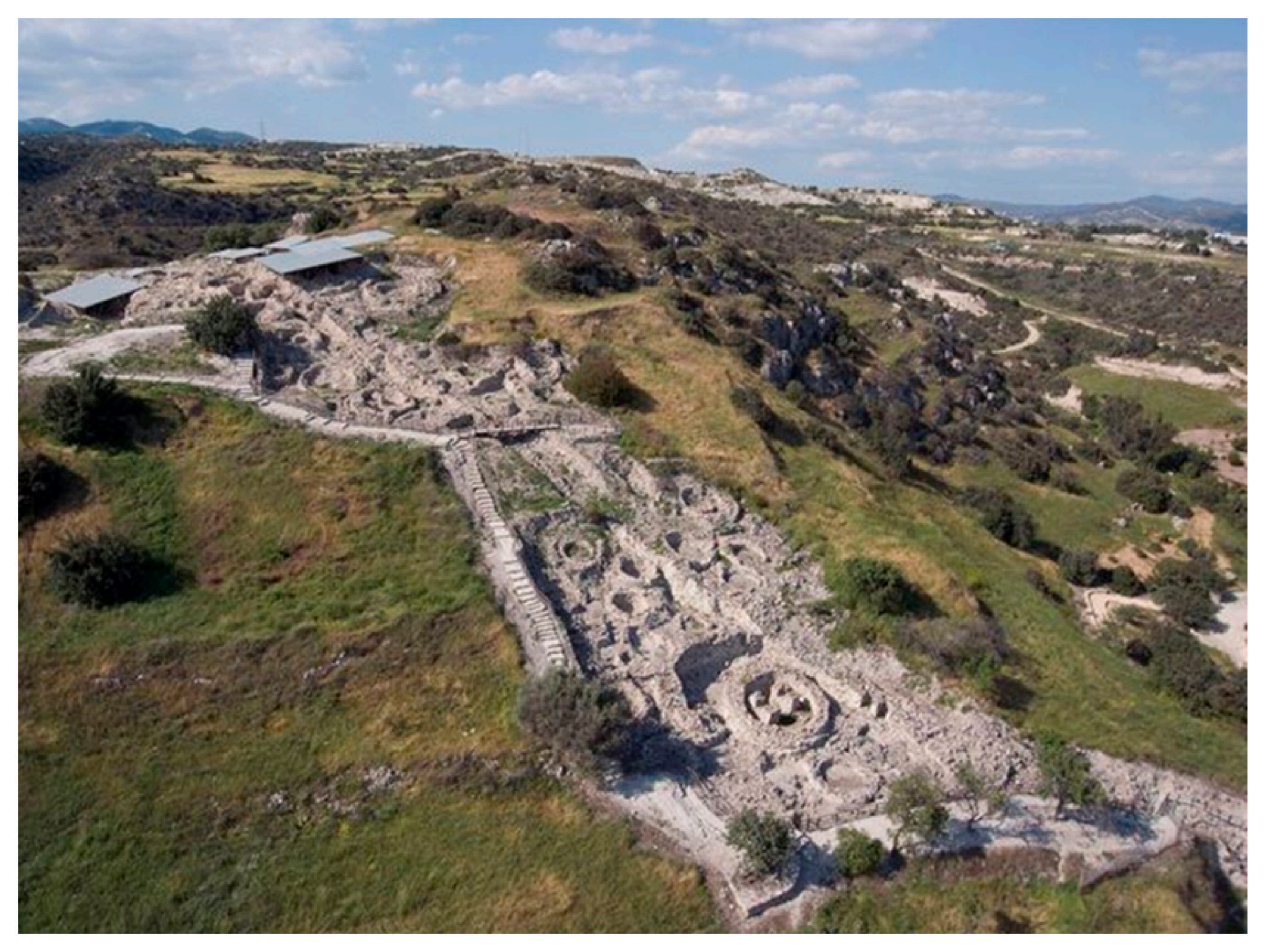
Choirokoitia UNESCO cultural heritage site.

The Neolithic settlement of Choirokoitia in Cyprus (Figure 4) is one of the most well-preserved prehistoric sites in the Mediterranean and offers an exceptional record of the Aceramic Neolithic period. First inhabited in the 8th millennium BC by settlers from the mainland, it developed into a flourishing community by the 7th millennium BC, marking the crucial shift from nomadic hunter-gatherers to settled farming societies. The site lies in today’s Larnaca district on the slope of a hill in the Maroni River valley, about six kilometres from the southern coast, a location that provided access to fertile land, water and coastal resources. Its circular stone-based dwellings, narrow passageways, and carefully arranged layout reveal a tightly organized community, while defensive walls point to collective protection and social cooperation. Excavations have uncovered evidence of early agriculture, domesticated animals and sophisticated tools, as well as burial practices within the settlement that reflect ritual and social complexity. As a UNESCO World Heritage site, Choirokoitia holds global archaeological importance, both for the insights it provides into early human civilization and for the reminder that such sites demand rigorous conservation to safeguard their legacy.

However, its geographical and geological context presents substantial conservation challenges due to climate change. Choirokoitia is situated within the Maroni River valley, near the Troodos Mountain range, where it is exposed to steep, sedimentary rock formations prone to weathering, erosion and slope instability. The region experiences long, dry summers with extreme heatwaves, followed by intense flash rainfall events during winter, which contribute to rockfalls and structural degradation. Rockfall is therefore a particular concern for both site conservation and visitor safety.

### 2.5. Smuszewo (Poland)

**Figure 5:**
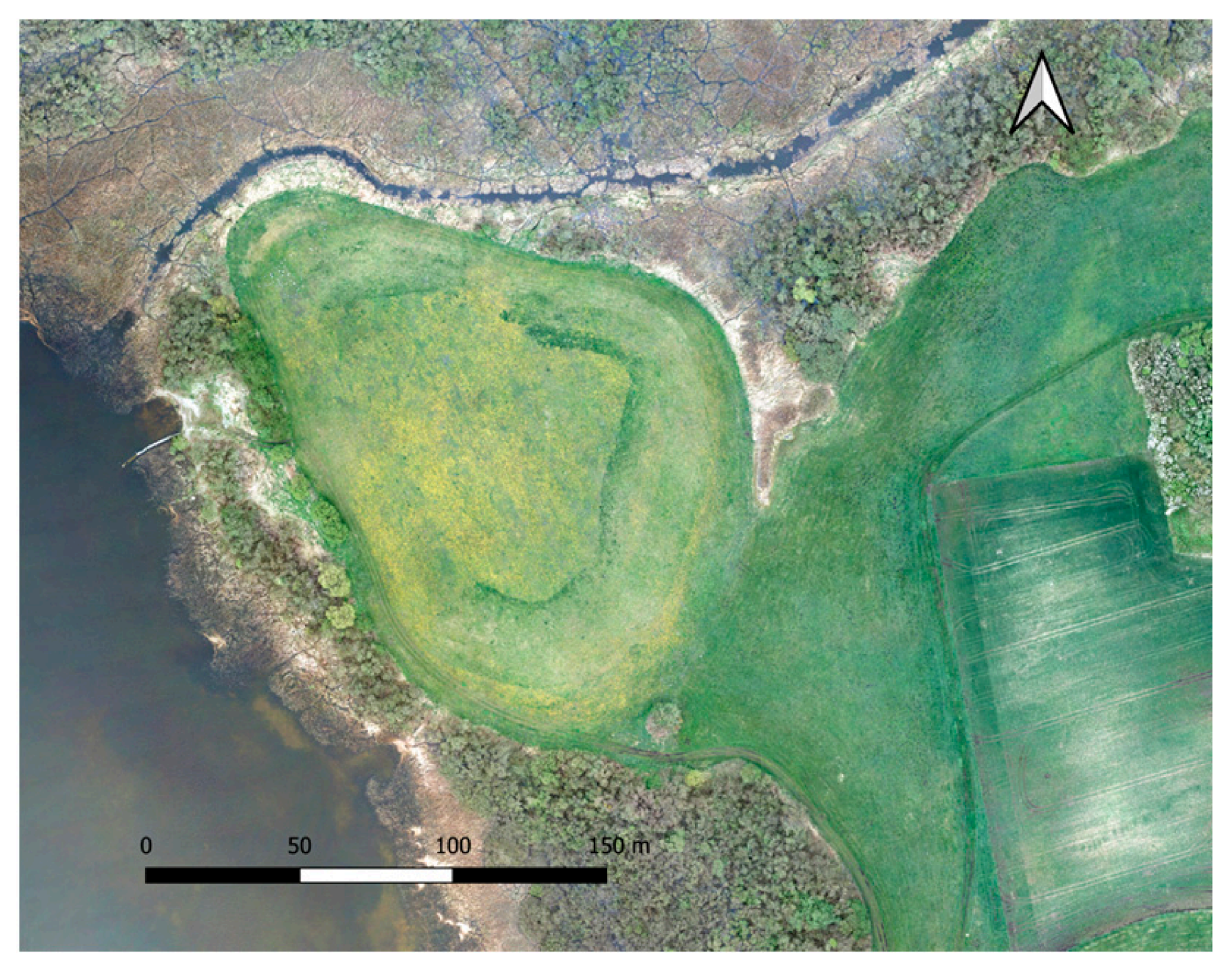
View of Smuszewo, site 3 from the air (orthomosaic, based on UAV survey carried out on 8 April 2024).

Smuszewo, site 3 (Figure 5), consists of relics of a fortified settlement from the Late Bronze Age/Early Iron Age (mid 8th century BC). These remains were identified in 1863 and originally referred to as the Czeszewo lake-dwelling, by analogy with lake-dwelling sites discovered shortly earlier in the Alpine region. The wooden relics were exposed during land reclamation at the base of a post-glacial valley and on the western shore of the lake. Lowered water levels revealed wooden breakwater structures in the peat–mineral valley fill [100] in the eastern part of the lake. Archaeological excavations conducted in the 1950s and 1960s confirmed the presence of wooden structures within the settlement, similar to those extensively excavated in Biskupin [101,102]. These wooden relics of internal buildings were located approximately 0.30–1.30 metres below the current ground level. Non-invasive investigations conducted in 2004 revealed a very clear plan of internal structures (wooden buildings, paved wooden streets, hearths, etc.) [102]. Several such settlements have been recorded in the Polish lowlands, and all of them face similar threats.

The land reclamation of the valley in the 19th century initiated the process of destruction of wood that ended up above the water level. Wooden relics require stable conditions, primarily constant humidity. The variability of the water level results from three main factors: (i) human activities (e.g., land reclamation), (ii) climate change-related trends (e.g., reduced precipitation), and (iii) seasonality (e.g., summer evaporation, lack of snow in winter). Monitoring reveals fluctuations in water levels and the associated threats. Complementary remote-sensing, meteorological, hydrochemical and geophysical observations were used to characterize environmental change trends, seasonal water-level variability and the preservation context of the buried archaeological remains.

### 2.6. Epidaurus (Greece)

**Figure 6:**
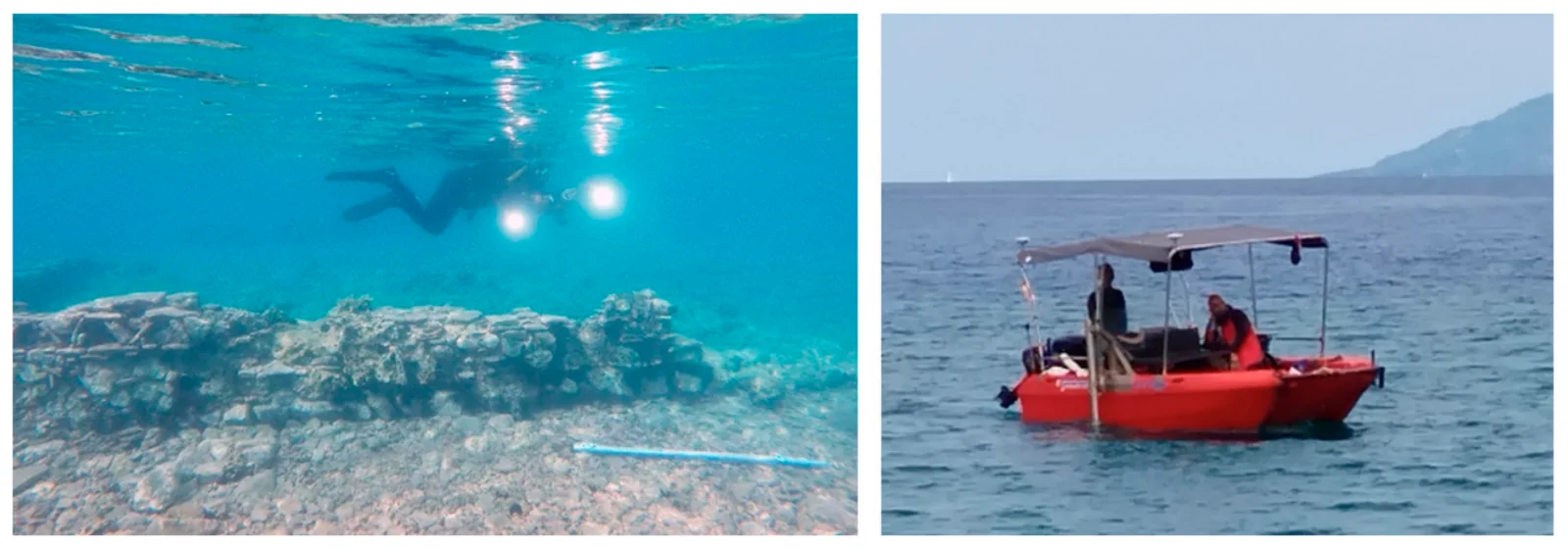
Fieldwork at the cultural heritage site of Ancient Epidaurus: underwater photogrammetric acquisition (**left**) and multibeam sonar survey (**right**).

The cultural heritage site of Ancient Epidaurus (Figure 6), situated in the bay of Agios Vlasios on the Peloponnese peninsula, comprises highly significant underwater and coastal remains dating back to the 12th century BC. Geographically, the site is located approximately 10 kilometres northeast of the world-renowned Sanctuary of Asklepios at Epidaurus, the famous main archaeological centre. Its most notable feature is the “Sunken City,” the submerged ruins of a Roman Villa, which lie in very shallow coastal waters.

This unique coastal and underwater environment faces a multitude of compounding threats that jeopardize its long-term survival. Bio-erosion from microorganisms and continuous water exposure gradually degrade the submerged structures. The visible coastal remains are endangered by the dual effects of sea level rise and coastal erosion, which are exacerbated by climate change. Furthermore, direct human activities, such as illegal mooring of tourist boats and coastal constructions, cause acute damage. Vandalism and potential seismic activity are also considered significant risks to the integrity of the antiquities.

At Epidaurus, the geometric documentation focuses on creating a detailed spatial record of the coastal and underwater archaeological resources. UAV photogrammetry, underwater photogrammetry, hydrographic multibeam echosounding and LiDAR-based measurements were applied to different parts of the coastal and underwater site according to their respective documentation requirements.

### 2.7. Roseninsel (Germany)

**Figure 7:**
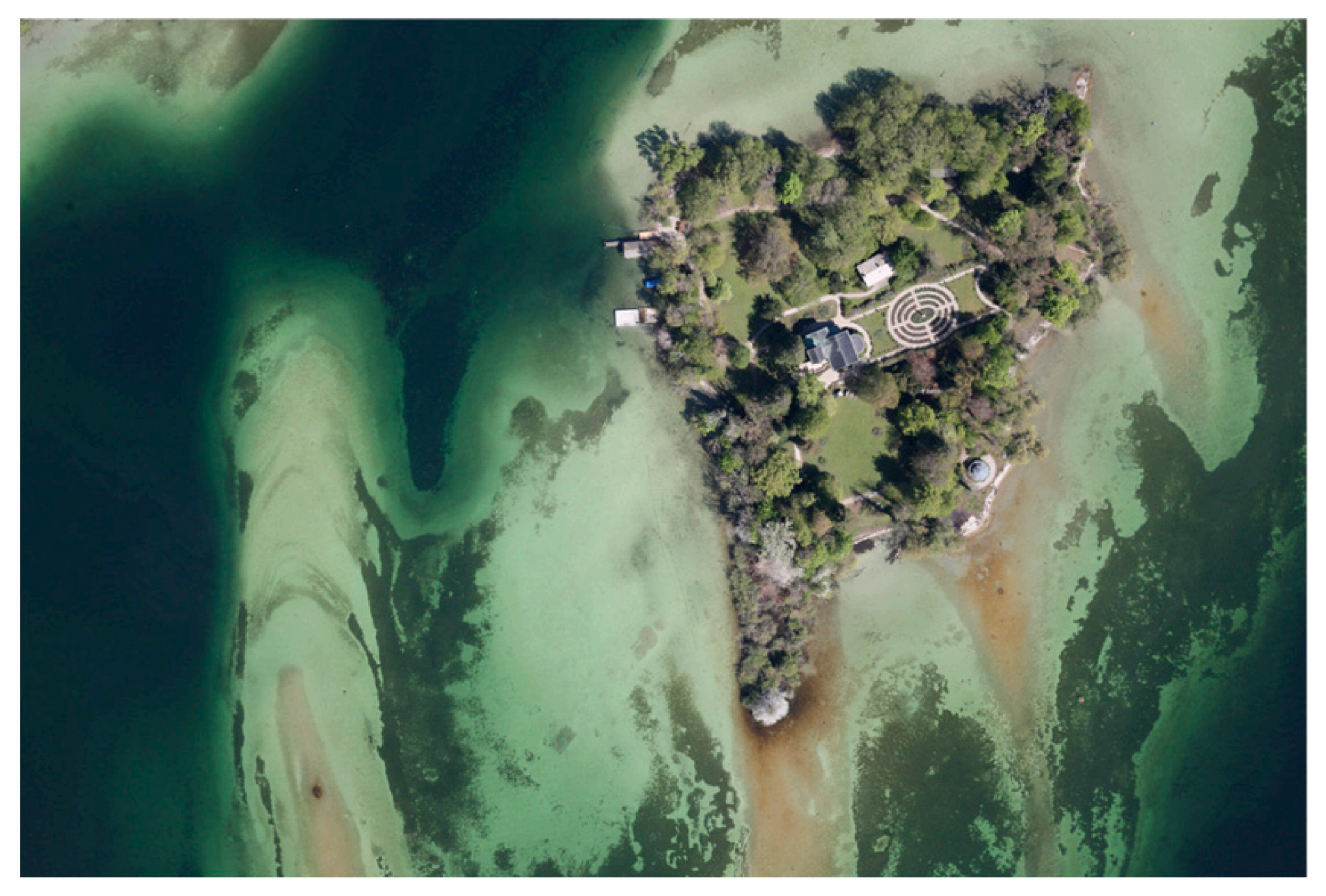
Aerial view of the Roseninsel cultural heritage site, acquired by the German Aerospace Center (DLR) 3K Camera System in April 2024.

Roseninsel (Rose Island) in Lake Starnberg (Figure 7) is located about 30 kilometres southwest of Munich. In the extensive shallow-water zone around the island, archaeological remains of prehistoric pile dwellings as well as wooden structures from the Early and Late Medieval periods are preserved, as the area had been repeatedly settled since the Neolithic period [103].

A single pottery shard, dating back to the Middle Neolithic period (approx. 4950–4500 BC), forms the first evidence of human activity on the island. A permanent presence on Roseninsel is attested by finds from the Late Neolithic period. Numerous artefacts, mostly found during the 19th century excavations, date to the Late Bronze Age. They indicate a heyday of human activity for this period. In addition to that, horizontal beams northeast of the island, dating to the early La Tène period, are the most recent pile dwelling structures known from the entire circum-Alpine region. Due to the rich remains of pile dwellings mentioned above, Roseninsel was inscribed as one of 111 sites in six countries that together form the UNESCO World Heritage site “Prehistoric Pile Dwellings around the Alps” in 2011.

While, for the Roman period, the use of the island is still under discussion, recent research has revealed unexpectedly extensive activity on the island during the Early Medieval period. This comprises the construction of a church as well as extensive bank reinforcements with slotted wooden posts and boards that date to the years around 700 AD according to dendrochronological research. After the destruction of the church and of the two Late Medieval bridges, which probably happened in the context of the Thirty Years’ War, a family of fishermen lived on the island. In the mid-19th century, the Bavarian royal family finally transformed Roseninsel into a summer destination, with a small pavilion-like castle, a rose garden and an English landscape garden.

The main problem for preservation is the erosion of the sediments covering the archaeological remains in the shallow water zone around the island, especially since the complex processes of erosion and re-sedimentation are not yet understood in detail. Nevertheless, monitoring data and comparisons of aerial photographs from past decades suggest that climate change is having an impact on erosion. The quagga mussel, which threatens archaeological sites such as Argilliez, was first detected in Lake Starnberg in late 2025 through environmental DNA (eDNA) analysis and was also identified morphologically in the summer of 2026, around 8 km south of Roseninsel. In addition, the falling lake level is endangering the timbers situated in very shallow water, especially when low water levels and frost coincide.

Existing information for Roseninsel included World Heritage monitoring data and results from the SuBoLakes research project [104]. However, neither a current nor a sufficiently accurate bathymetric dataset was available for the waters around Roseninsel. To address this gap, broader sonar-based bathymetric coverage was acquired across the core and buffer zones of the UNESCO World Heritage property, while high-density underwater imagery over a localized area containing archaeological remains enabled the generation of a high-resolution structure from motion (SfM) and multi-view stereo (MVS) model of the pile dwelling remains.

### 2.8. Les Argilliez (Switzerland)

**Figure 8:**
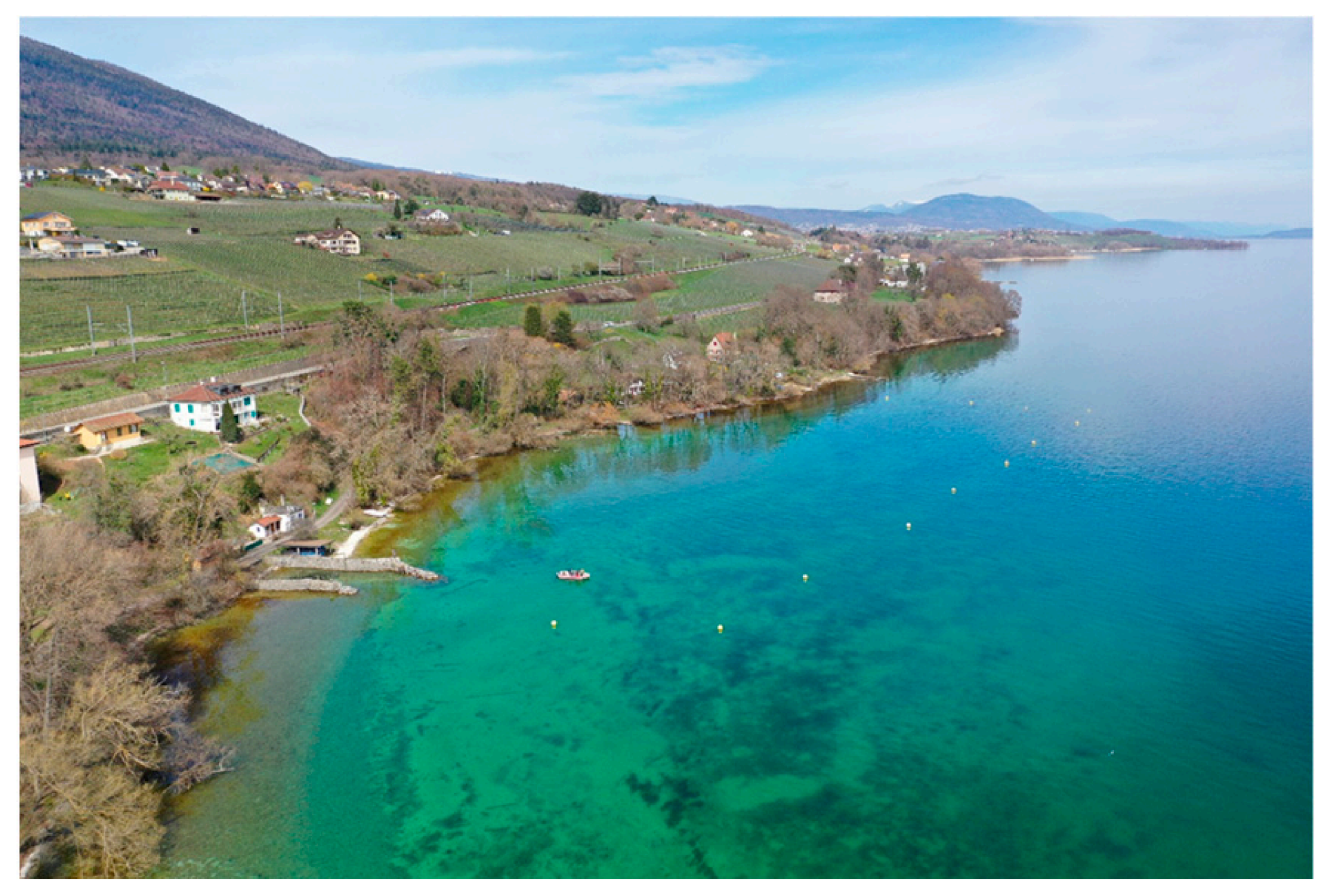
Aerial view of the Argilliez site, protected by yellow buoys that prohibit navigation within this UNESCO-listed area.

This large pile dwelling of Argilliez (Figure 8) is located in the bay of Gorgier on the north shore of Lake Neuchâtel in Switzerland and is listed as part of UNESCO’s World Heritage. In the 1990s, eight samples of wooden piles were taken; their dendrochronological dating suggests the construction of two successive villages in the fourth millennium BC. The Argilliez site is a protected site, forbidden to navigation for some years and entirely marked by yellow buoys. It is located at a depth of 2–3 m and is covered almost entirely by a layer of pebbles of anthropic and natural origin. In 2014, a topographic survey campaign confirmed a larger spatial amplitude. Indeed, at the end of this operation, 4834 piles were identified and densely distributed over a length of 140 m, parallel to the shoreline for a width of 50 m (7000 m^2^). Within this zone, two zones can be distinguished: to the west, the piles are barely perceptible, very tightly packed and protected by a dense accumulation of pebbles; whereas to the east, they are apparent, sometimes up to nearly 50 cm high, and scattered in the lacustrine chalk. This differentiated preservation of the two zones is probably based on the contiguous location of the two villages: the older one, built in the classical Cortaillod, would be followed to the west by the later Cortaillod, better preserved today from erosion.

Off the villages, to the east and west, an erosion front is active and progressively cuts into the site. It comes from a notable difference in depth between the centre of the site and the periphery, which allows lake currents to progressively erode the deposit from the periphery. This front is less active than those observed in other bays of the lake, but it must be constantly monitored. The currents in the lake are very dynamic, and their intensity can change from year to year. Regular 3D modelling of the erosion front is essential to monitor the evolution of the erosion and to allow, if necessary, to put in place measures to physically preserve this World Heritage Site. The above-ground height of the piles of a village is also an indicator of the evolution of erosion. The more the piles protrude from the sediment, the more intense the erosion. It is also necessary to follow the evolution of this phenomenon, year after year. Previous photogrammetric monitoring was limited to control areas; site-wide 3D documentation is therefore needed to evaluate pile exposure and erosion patterns across the archaeological area.

Global warming with a decrease in precipitation has resulted in a lower average lake level, which has accentuated the erosive phenomenon. The water quality has also been changing for several years with less phytoplankton and water that is becoming clearer. A proliferation of lake mussels is visible in the western half of the lake and is moving eastward. The Argilliez site is already affected by this invasion. These mussels can contribute to the degradation of archaeological material, particularly organic remains.

At Argilliez, geometric documentation focuses on recording the submerged pile-dwelling landscape and its erosion-related preservation context. Site-wide underwater photogrammetry provides a detailed 3D representation of the archaeological area, while flash LiDAR provides localized active-ranging observations of selected pile-dwelling remains. Environmental observations of lake level, water quality and biological activity provide additional context for interpreting the processes affecting site preservation.

## 3. Materials and Methods

This section presents the geometric documentation activities undertaken within TRIQUETRA for the eight pilot cultural heritage sites. We report both airborne and underwater acquisitions and present the resulting 2D, 2.5D and 3D outputs, including dense point clouds, textured meshes, orthomosaics, DSMs/DTMs, bathymetric products and underwater LiDAR point clouds. To facilitate comparison among the heterogeneous survey configurations, Table 2 summarizes the conservation questions and monitoring indicators, survey dates and acquisition configurations, sensor and platform specifications, georeferencing and independent validation strategies, processing software and output resolution across the eight pilot sites. For clarity, the geometric quality indicators reported in this section and summarized in Table 2 are distinguished according to their meaning. Ground sampling distance (GSD) and model/output resolution describe the spatial sampling or level of detail of the generated products and are not measures of positional accuracy. Ground control point (GCP) residuals quantify the fit of control points used in georeferencing and are therefore not interpreted as independent estimates of product accuracy. Independent positional accuracy is reported only where separate check points (CPs) or independent reference measurements were available. Where applicable, residuals and independent positional accuracy are reported as root mean square error (RMSE), with horizontal (XY), vertical (Z) and three-dimensional (3D) components provided where available. Sensor-specific precision values, such as the flash LiDAR intrinsic equivalent in-water range precision, refer to intrinsic measurement precision and are reported separately from positional accuracy.

### 3.1. UAV-Based Photogrammetric Documentation

This subsection details the UAV surveys conducted at the cultural heritage sites of Kalapodi, Ventotene, Aegina, Choirokoitia, Smuszewo and Epidaurus.

#### 3.1.1. Kalapodi

The primary objective of the geometric documentation of the sanctuary at Kalapodi was to capture the site’s structural details at high geometric detail, creating a baseline dataset to support the analysis of material deterioration from environmental factors, particularly the recurring threat of frost damage. A DJI Matrice 300 real-time kinematic (RTK) UAV was used for the photogrammetric survey, equipped with a DJI P1 full-frame 45-megapixel camera and a 35 mm lens. The data acquisition campaign was conducted in June 2024 and involved two distinct flights: one autonomous, grid-pattern flight at approximately 40 m above ground level (AGL) that captured 810 vertical images for overall coverage and a second manual flight at approximately 10–15 m AGL that collected 472 nadir and oblique images to ensure detailed modelling of vertical structures. To support georeferencing, a ground control network was established; 13 GCPs were measured using a Leica TS10 1″ total station (Leica Geosystems AG, Heerbrugg, Switzerland), building upon two base points established with an EMLID global navigation satellite system (GNSS) receiver (Emlid Tech Kft., Budapest, Hungary).

**Figure 9:**
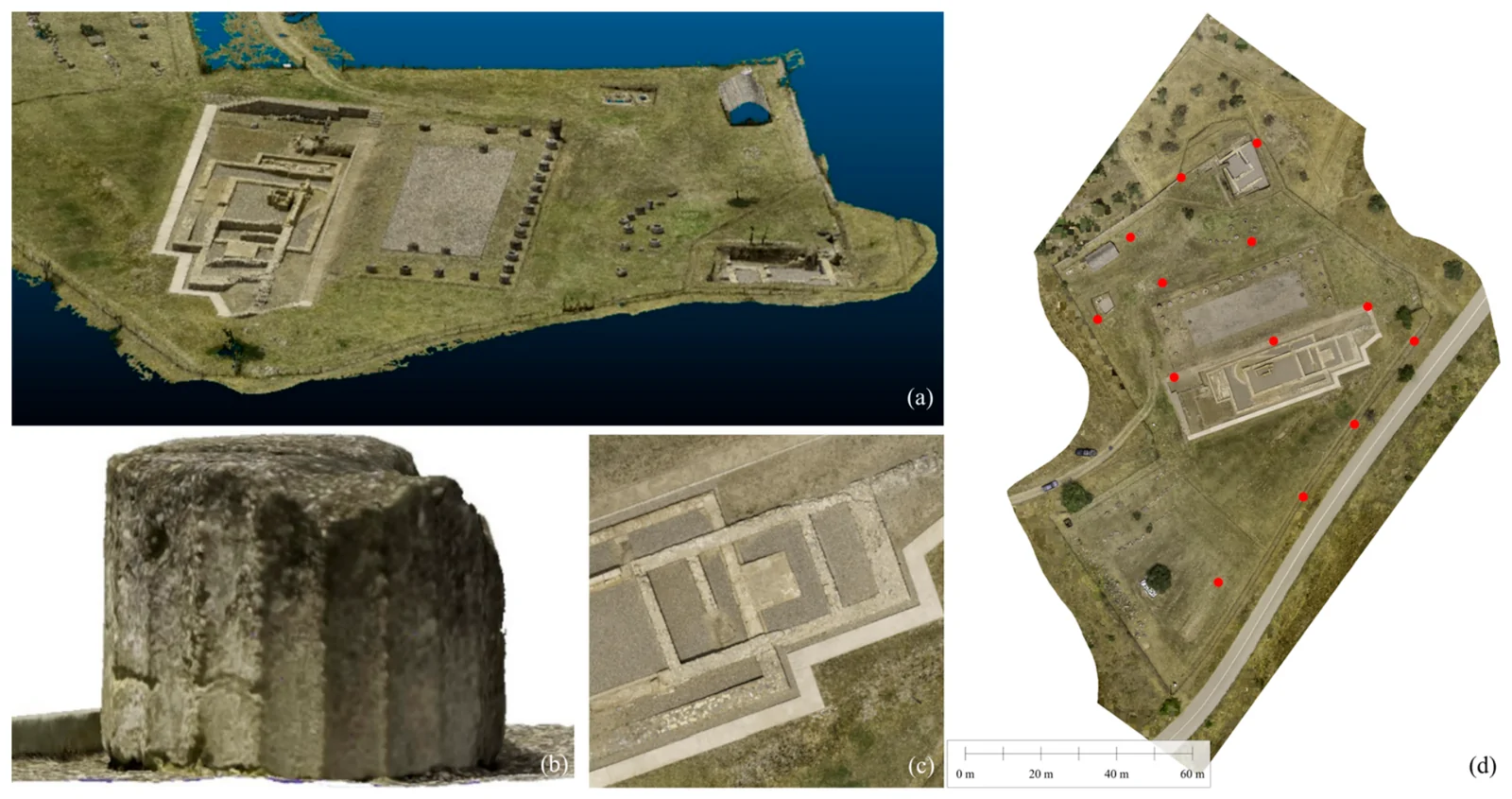
Geometric documentation of the Kalapodi sanctuary: (**a**) general view of the textured 3D model; (**b**) detailed view of the 3D model; (**c**) detailed view of the orthomosaic; (**d**) orthomosaic showing the spatial distribution of the 13 ground control points (GCPs, red circles) used for georeferencing.

The dataset of 1282 images was processed using the RealityCapture software, now rebranded as RealityScan [105], a choice informed by previous comparative analyses of 3D modelling packages for complex heritage geometries [106]. The SfM process resulted in a sparse point cloud of 3.2 million points. Following integration of the GCPs, the final alignment yielded an overall GCP residual RMSE of approximately 3 mm. No independent CPs were withheld for validation of the final model. The subsequent reconstruction produced a detailed 3D model of 1 billion triangles, which was later simplified to 50 million triangles for practical application. The final textured 3D model and the derived orthomosaic, which features a GSD of 2 mm, provide a high-resolution georeferenced record of the site’s condition. A DSM with a spatial resolution of 0.5 m was also generated. Figure 9 presents the resulting 3D model, derived orthomosaic and spatial distribution of the GCPs.

#### 3.1.2. Ventotene and Santo Stefano

A UAV-based photogrammetric survey was performed in Ventotene in March 2023 to generate a high-resolution 3D reconstruction of the Punta Eolo promontory and the archaeological site of Villa Giulia. A DJI Matrice 300 equipped with a DJI P1 full-frame 45 MP camera and a 35 mm lens was operated at a flight height of approximately 50 m to collect 855 nadir and oblique images. While nadir images ensured complete coverage of the study area, oblique images were essential for accurately capturing the vertical geometries of the archaeological structures and, critically, the surrounding sea cliffs, which represent a key element of interest in the area for geomorphological and slope stability analyses.

We used nine GCPs strategically distributed across the site to support georeferencing. The GCPs were measured with a high-precision GNSS receiver in WGS84/UTM zone 33N. Georeferencing yielded GCP residual RMSE values of 0.6 cm horizontally and 0.93 cm vertically, corresponding to a 3D RMSE of 1.1 cm. No independent CPs were withheld for validation of the final model.

**Figure 10:**
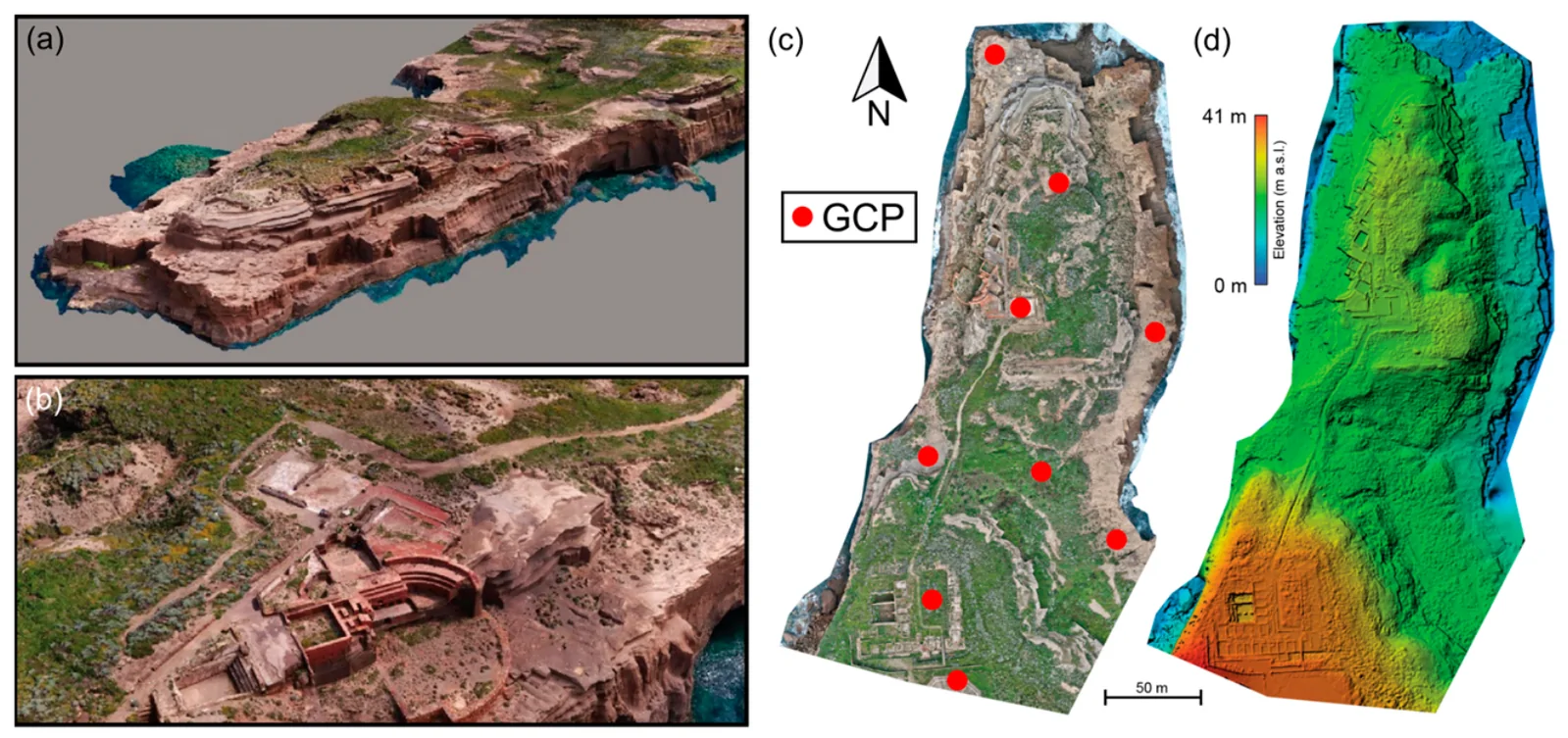
UAV-based 3D documentation of the Punta Eolo promontory (Ventotene Island), where the archaeological remains of Villa Giulia are located. (**a**) Oblique perspective view of the reconstructed 3D textured model of the promontory, showing the steep coastal cliffs and the close spatial relationship between the archaeological site and the plunging sea cliffs. (**b**) Detail of the Villa Giulia complex and adjacent cliff edges; (**c**) UAV-derived orthomosaic of the entire promontory, showing the spatial distribution of the nine GCPs used for georeferencing. (**d**) Digital surface model (DSM) generated from the photogrammetric reconstruction, with elevation values ranging from sea level to ~41 m above sea level.

The photogrammetric processing followed a standard SfM–MVS workflow implemented in Agisoft Metashape Professional 2.0.1 [107]. Camera interior and exterior orientation parameters were estimated during the SfM stage, resulting in the generation of a sparse point cloud of approximately 1 million points. A dense point cloud was subsequently reconstructed, comprising approximately 56 million points, which provided a highly detailed 3D representation of both the archaeological remains and the coastal morphology. Surface reconstruction generated a textured 3D mesh model comprising approximately 500,000 vertices and 1 million faces, resulting in a photorealistic representation of the site (Figure 10a,b). From the processed dataset, we also produced a high-resolution orthoimage with a GSD of 1 cm and a DSM at 5 cm resolution (Figure 10c,d). The orthomosaic of Figure 10c also shows the spatial distribution of the GCPs used for georeferencing. The final products cover an area of approximately 121,562 m^2^ with an average ground resolution of 8.99 mm per pixel. These datasets provide a geometric reference for site documentation and for separate hazard analyses undertaken within TRIQUETRA.

#### 3.1.3. Aegina

A UAV-based photogrammetric campaign was implemented in the archaeological site of Aegina Kolonna to generate a high-resolution 3D representation of the site. The primary image acquisition was carried out with a DJI Mavic 3 Enterprise drone, equipped with a 20 MP camera with a 12.29 mm focal length, which captured approximately 950 nadir images for complete site coverage, acquired at a flight height of 40 m. Furthermore, in order to ensure that architectural details of walls, the slopes and other vertical elements were properly captured, an additional set of 4900 oblique images was acquired from low-altitude flight paths at flight heights ranging from 15 to 40 m. Finally, a supplementary set of 930 images was also collected using a DJI Mavic 2 Pro drone equipped with a 20 MP camera with a 10.26 mm focal length to enhance coverage of peripheral areas.

Ground reference data were collected using a Leica 1200 GNSS receiver operating in GGRS87, with corrections from the MetricaNET reference station network delivered via Networked Transport of RTCM via Internet Protocol (NTRIP). A total of 24 targets were surveyed across the site. Among these, 14 were used as GCPs for georeferencing, while the remaining 10 were withheld from the adjustment and were used as independent CPs for accuracy assessment.

**Figure 11:**
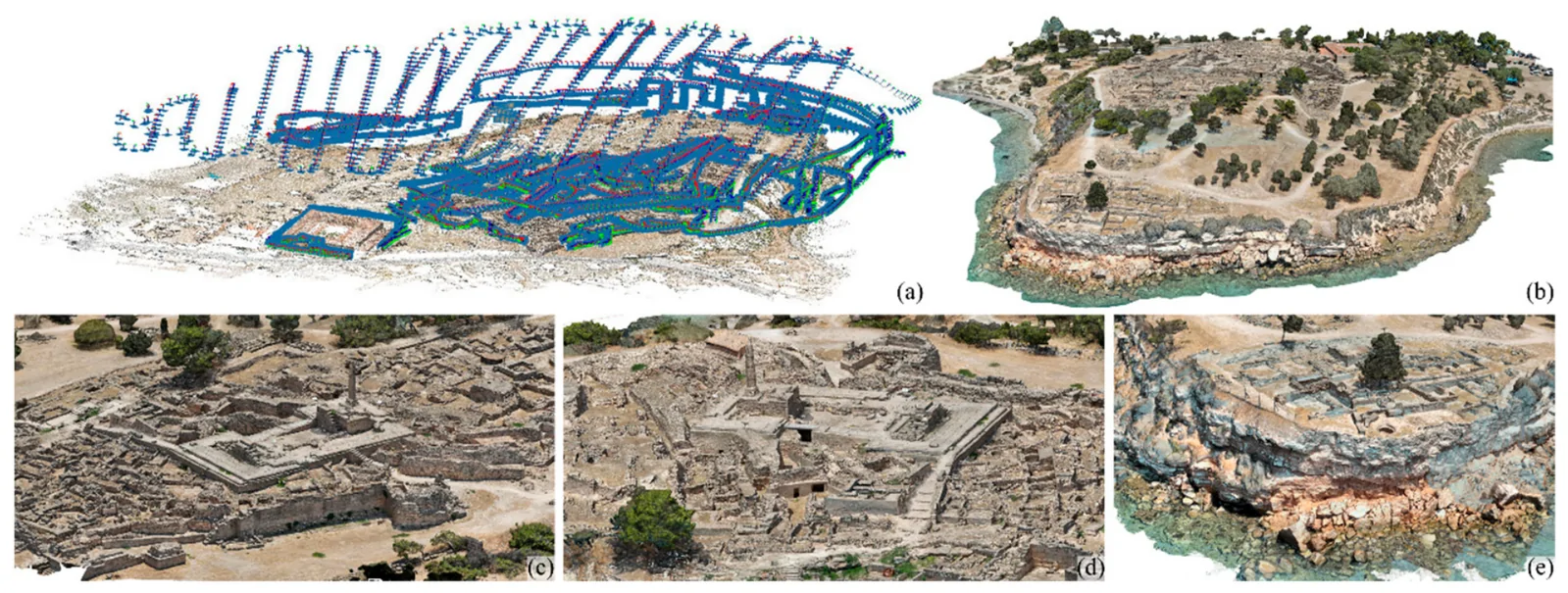
(**a**): Structure from motion (SfM) results: oriented images and sparse 3D point cloud of the archaeological site of Aegina Kolonna; (**b**): 3D dense point cloud; (**c**–**e**): zoom-in views of the 3D dense point cloud.

**Figure 12:**
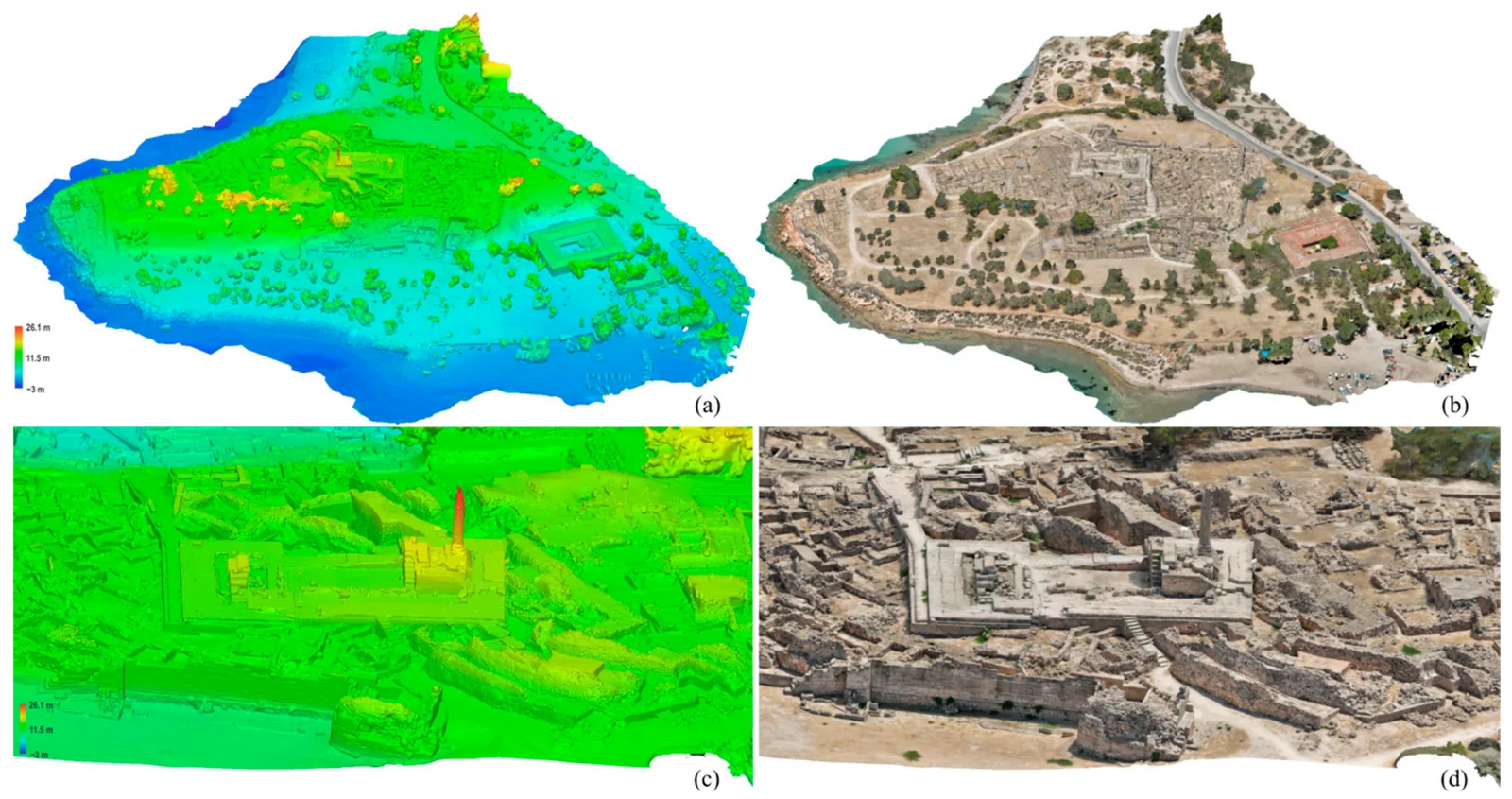
Views of the 3D model of the Aegina Kolonna archaeological site. (**a**): Elevation-coloured 3D mesh model of the entire site, visualised using a height-based colour ramp (−3 m to 26.1 m); (**b**): textured 3D mesh model of the entire site; (**c**): detail view of the elevation-coloured mesh; (**d**): detail view of the textured mesh.

**Figure 13:**
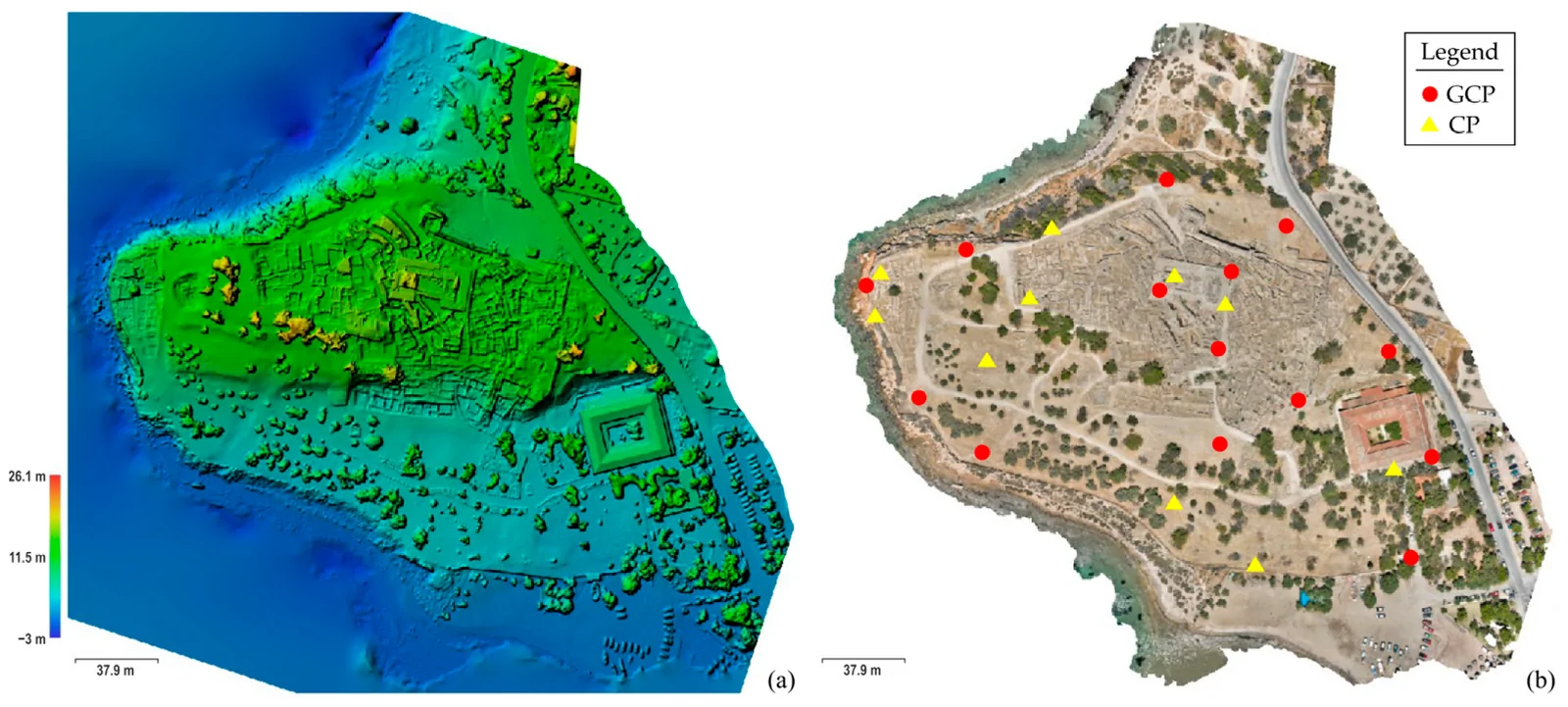
Geometric documentation products and GCP/CP distribution at Aegina Kolonna: (**a**) DSM of the archaeological site; (**b**) orthomosaic showing the spatial distribution of the 14 GCPs (red circles) used for georeferencing and the 10 independent CPs (yellow triangles) used for accuracy assessment.

Photogrammetric processing followed a standard SfM–MVS workflow using Agisoft Metashape Professional 2.2.2. The SfM process resulted in the estimation of the camera interior and exterior orientation parameters and the production of a sparse point cloud of approximately 2.4 million points (Figure 11a). Georeferencing was performed using the 14 GCPs, yielding horizontal and vertical residual RMSE values of 4.6 cm and 3.0 cm, respectively, with a 3D RMSE of 5.5 cm. Independent validation at the 10 CPs resulted in an RMSE of 3.5 cm in the horizontal component and 3.3 cm in the vertical component, corresponding to a 3D RMSE of 4.8 cm. Dense reconstruction followed, producing a dense point cloud of roughly 61 million points (Figure 11b–e). Surface reconstruction was the next step, producing a detailed 3D mesh with approximately 9.9 million vertices and 19.8 million faces and texture mapping was then applied to the produced 3D mesh, using the oriented images (Figure 12). A high-resolution DSM was also derived at a 5 cm spatial resolution. Additionally, high-resolution orthomosaics were generated with GSDs of 1 cm and 2.5 cm. Figure 13 presents the DSM and orthomosaic, with the latter also showing the spatial distribution of the 14 GCPs used for georeferencing and the 10 independent CPs used for accuracy assessment across the surveyed area.

#### 3.1.4. Choirokoitia

To establish a spatial baseline for future site monitoring, geometric documentation of the Choirokoitia site was carried out through UAV-based photogrammetry combined with RTK GNSS measurements of ground reference points. A DJI Mavic 3 Enterprise drone equipped with a 20 MP camera was used to capture imagery during repeated survey periods in June 2023 and May, October and November 2025. These datasets were processed to produce georeferenced DSMs and orthomosaics that provide reference products for future temporal comparison, following methodologies described in ref. [108].

The UAV was operated at an altitude of 40 m above the highest elevation of the site, with a flight speed of 6 m/s to ensure stable image acquisition. Each mission generated approximately 450 overlapping images. A network of 30 surveyed ground points, measured using a dual-frequency GNSS receiver, was distributed across the site to support georeferencing and independent accuracy assessment, which was especially important due to the steep 50 m slope of the hill where the settlement is located. Of these points, 25 were used as GCPs and 5 were withheld as independent CPs. The processed datasets produced orthomosaics with a GSD of 5 cm and DSMs with a spatial resolution of 10 cm.

Data were referenced to the Cyprus Local Transverse Mercator projection (LTM CGRS93) with the Cyprus Geodetic Reference System 1993 datum and the WGS84 ellipsoid. Prior to photogrammetric processing, lens distortions were corrected. Image datasets were then processed in Agisoft Metashape Professional 2.2.2. The workflow involved aligning images, generating dense point clouds, producing orthomosaics and creating DSMs. From these, contour maps and elevation models were derived. The photogrammetric adjustment yielded GCP residuals of RMSE XY = 4 cm, RMSE Z = 3 cm and RMSE 3D = 5 cm. Independent validation at the 5 CPs resulted in RMSE XY = 3 cm, RMSE Z = 8 cm and RMSE 3D = 9 cm.

**Figure 14:**
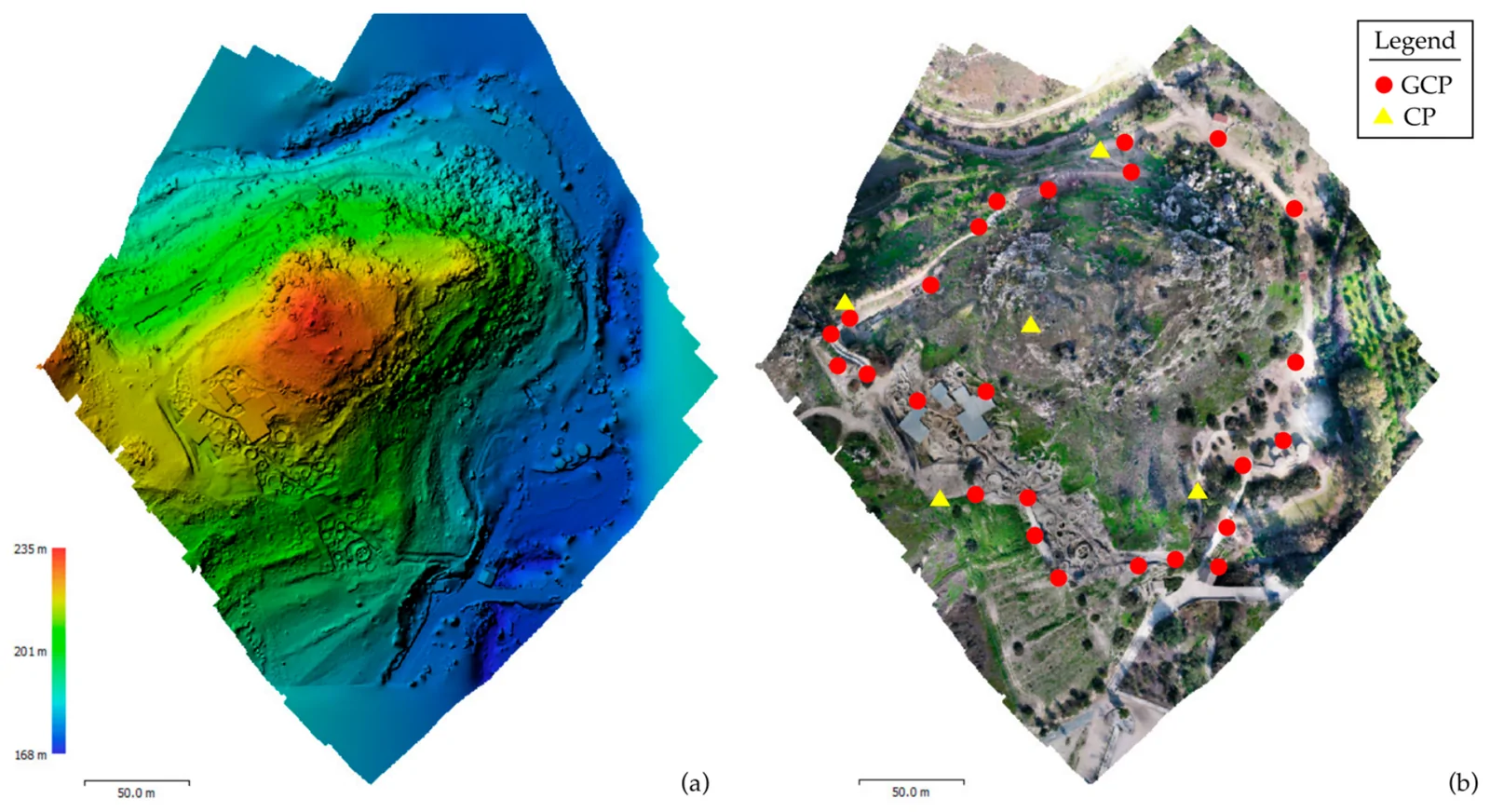
Geometric documentation products and GCP/CP distribution at Choirokoitia: (**a**) digital surface model (DSM) of the archaeological site; (**b**) orthomosaic showing the spatial distribution of the 25 ground control points (GCPs; red circles) used for georeferencing and the 5 independent check points (CPs; yellow triangles) used for accuracy assessment.

The resulting dense point clouds, supported by the distributed GCPs, provided detailed georeferenced 3D reconstructions of the site. These products provide repeated spatial records of the Choirokoitia settlement and its surrounding terrain, serving as a basis for future assessment of morphological changes through inter-epoch comparison. Figure 14 presents the DSM and orthomosaic of the Choirokoitia site, with the orthomosaic also showing the spatial distribution of the GCPs and the CPs.

#### 3.1.5. Smuszewo

Three-dimensional models of archaeological sites are an essential management tool, particularly for monitoring their preservation state and environmental or anthropogenic changes. Across Poland, publicly available data obtained through airborne laser scanning and regularly conducted aerial photography (vertical) allow for the creation of models with a resolution of about 0.5 m. While these datasets are undoubtedly useful, they do not enable the creation of more detailed imagery or frequent monitoring of archaeological sites.

For this reason, as part of the TRIQUETRA project, over 30 UAV flights were conducted at the Smuszewo site, enabling the generation of orthomosaics, DSMs and 3D models from repeated survey epochs and their multi-temporal comparison. The flights were carried out using Yuneec H520E drones with precise registration of the projection centre position using RTK corrections obtained from ASG-EUPOS ground stations via a mobile network (Active Geodetic Network EUPOS). The drone was equipped with an E90X red–green–blue (RGB) camera and/or an E10T thermal sensor (Yuneec Europe GmbH, Kaltenkirchen, Germany). Flights were conducted at speeds of up to 3–4 m/s, which, with the correction network, theoretically allows for projection centre accuracy of up to 3 cm horizontally and about 5 cm vertically. However, due to the distance from reference stations (ASG-EUPOS network), the presence of numerous wind turbines in the area, and regional GNSS signal interference, the achieved RTK camera-centre positioning accuracy was around 10–12 cm. Flights were conducted at altitudes ranging from 30 to 120 m AGL with 85% forward overlap and 60% side overlap.

**Figure 15:**
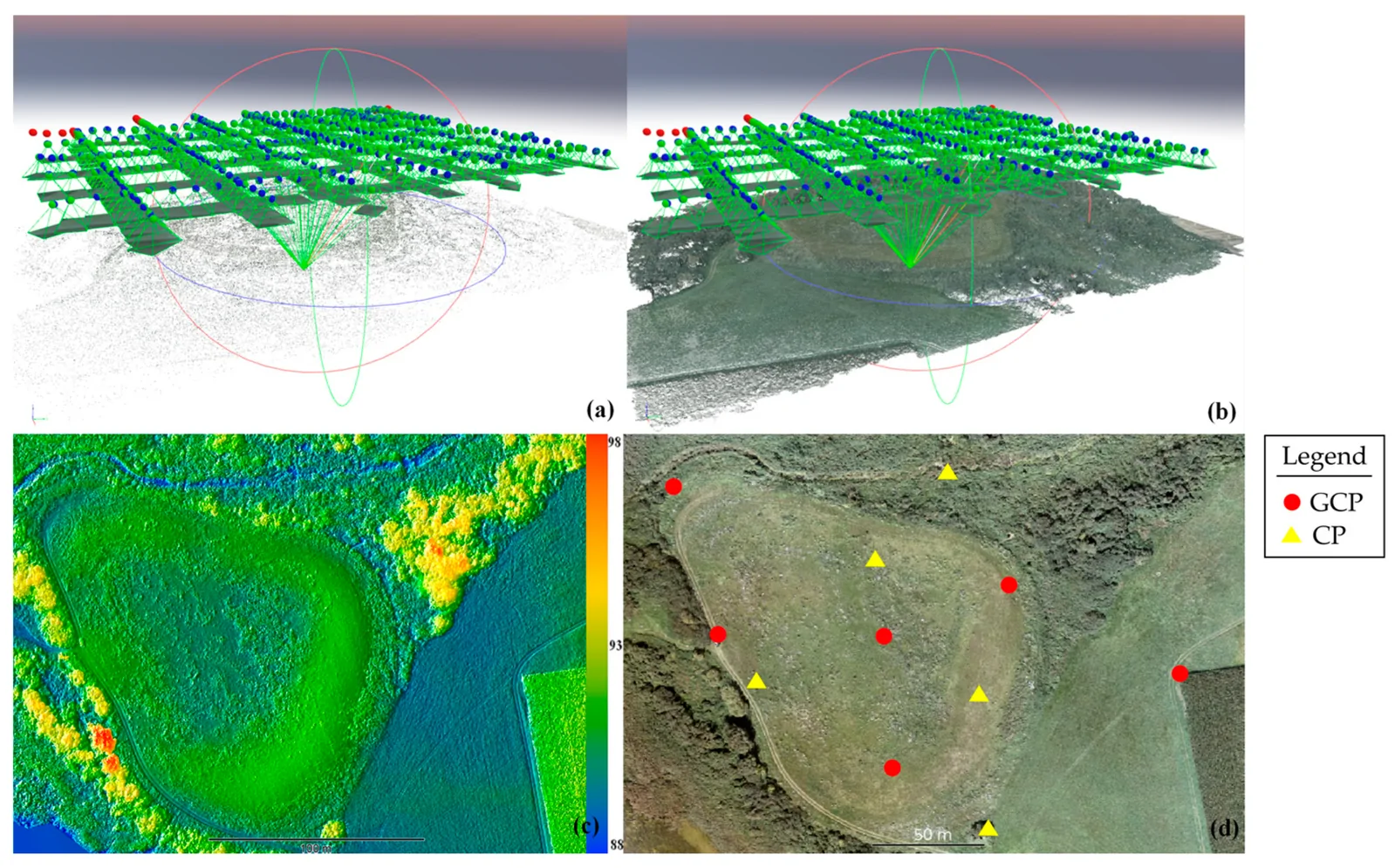
Smuszewo, site 3. Products of the UAV survey carried out on 22 August 2025: (**a**) aligned photos and tie points cloud, (**b**) dense point cloud, (**c**) DSM, (**d**) orthomosaic with GCPs (red circles) and CPs (yellow triangles) marked.

Photogrammetric processing was performed using Pix4Dmapper, version 4.9 [109]. The modelling strategy involved auto-calibration of the Brown distortion model (including three radial and two tangential distortion coefficients) with a locked focal length. This approach ensured high consistency of results, verified using GCPs and independent CPs. Across the survey epochs, GCP residuals were approximately 1.2–2.5 cm in XY and 2.3–3.0 cm in Z, while independent CP accuracy was approximately 2–3 cm in XY and 4 cm in Z. To improve modelling accuracy, we employed two complementary strategies: (i) flight plans with a grid of perpendicular flight lines over the area and (ii) image sets acquired at multiple altitudes (e.g., 60 m and 90 m or 90 m and 120 m AGL). During the automatic image alignment stage, both manual and automatic verification of tie point sets was applied. Depending on project needs, products were generated at different spatial resolutions and levels of detail: point clouds (up to 2 billion points), 3D triangulated irregular network (TIN) textured models, standard orthomosaics with GSDs of 1–3.5 cm (depending on flight altitude between 30 and 120 m AGL), and DSM/DTM products with spatial resolutions as fine as approximately 1 cm (Figure 15).

**Figure 16:**
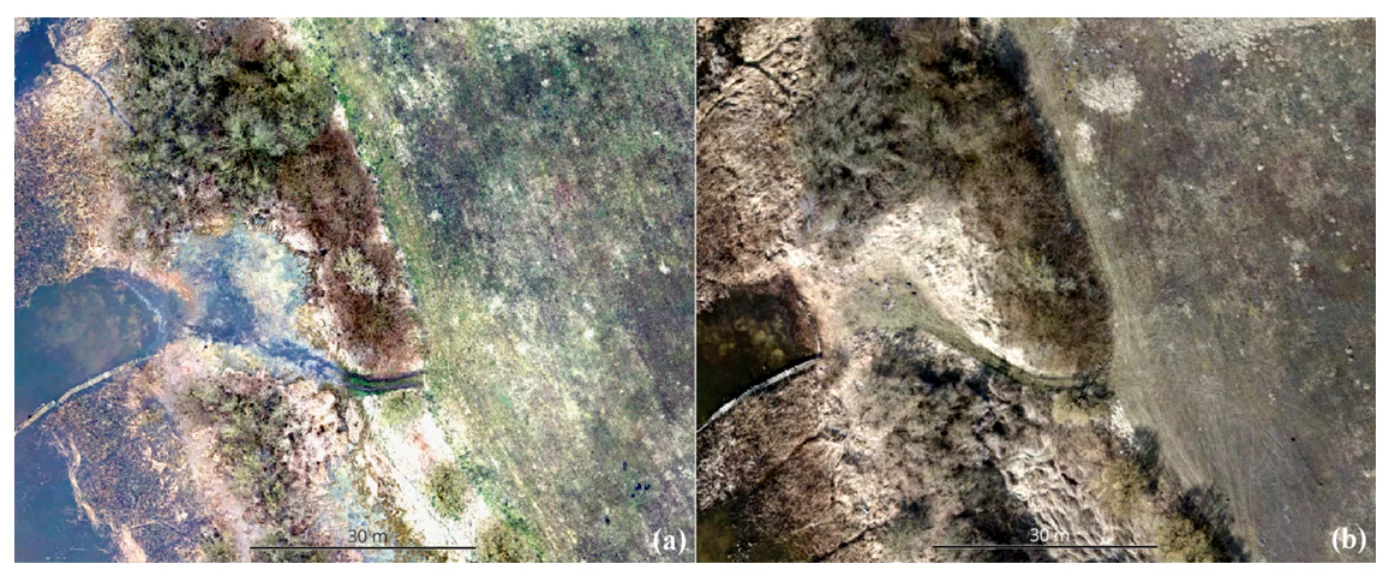
Smuszewo, site 3. Orthomosaics presenting changes of water level in the lake: (**a**) 13 March 2024; (**b**) 10 March 2025.

**Figure 17:**
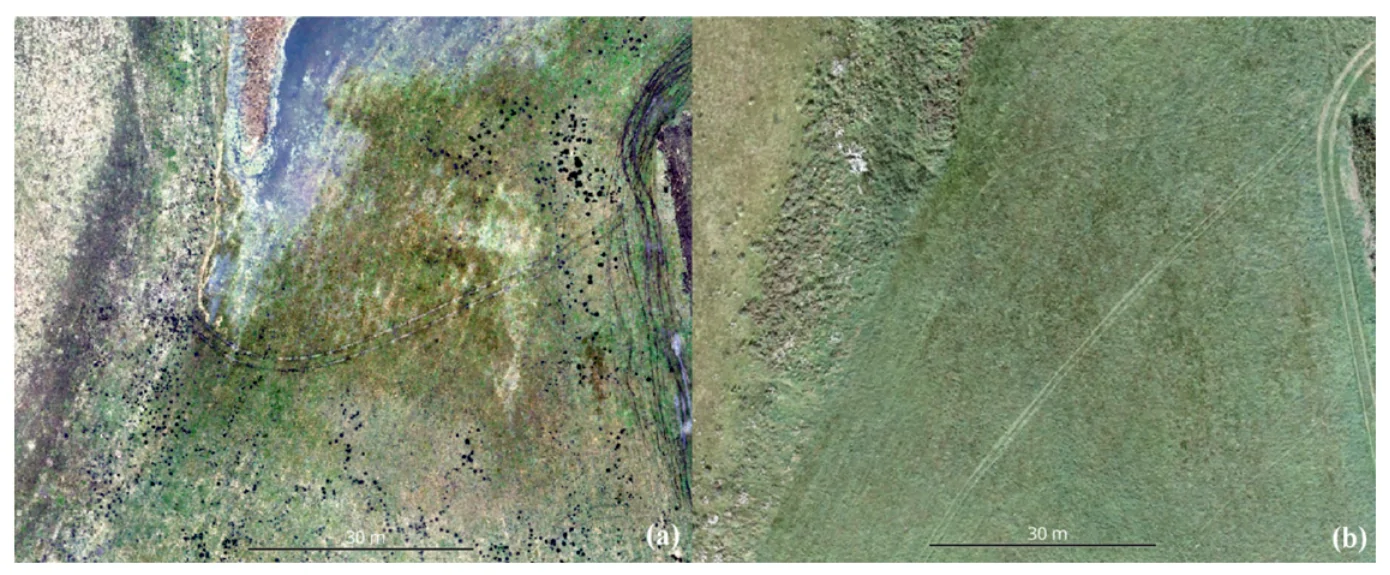
Smuszewo, site 3. Orthomosaics illustrating traces of mole activity in the same place but under different conditions: (**a**) 13 March 2024; (**b**) 22 August 2025.

The repeated datasets were used to observe changes in the lake’s coastal zone, including variations in water level. The repeated orthomosaics and surface models enabled the observation of seasonal water-level variations (Figure 16). It was also possible to identify areas in the reed belt where vegetation changes occurred during the documentation period (e.g., trees dried out or were cut down, animal paths appeared and disappeared). Within and near the site, mole activity variability (presence and distribution of molehills) was also observed, likely serving as an indirect indicator of water level and soil moisture fluctuations (Figure 17).

#### 3.1.6. Epidaurus

The aerial photogrammetric survey of the expansive coastal area of Ancient Epidaurus was conducted in May 2024 to create a detailed georeferenced 3D model of the terrain and the archaeological excavations. This work follows established photogrammetric principles for heritage documentation, including robust SfM–MVS workflows [110].

The primary equipment was a DJI Matrice 300 RTK UAV. A total of seven flights were performed to comprehensively capture the large and topographically varied site. Five of these were autonomous flights covering the wider coastal zone, capturing 4949 vertical images and were conducted at approximately 45 m AGL; three of these flights strategically utilised a terrain-follow function to accommodate the significant height differences in the landscape and maintain a consistent resolution. To document the ongoing excavations in greater detail, a multi-scale approach was adopted, similar to frameworks proposed for damaged heritage sites [111]. Two additional manual flights were conducted at approximately 10–15 m AGL, adding 668 vertical and oblique images to the dataset.

**Figure 18:**
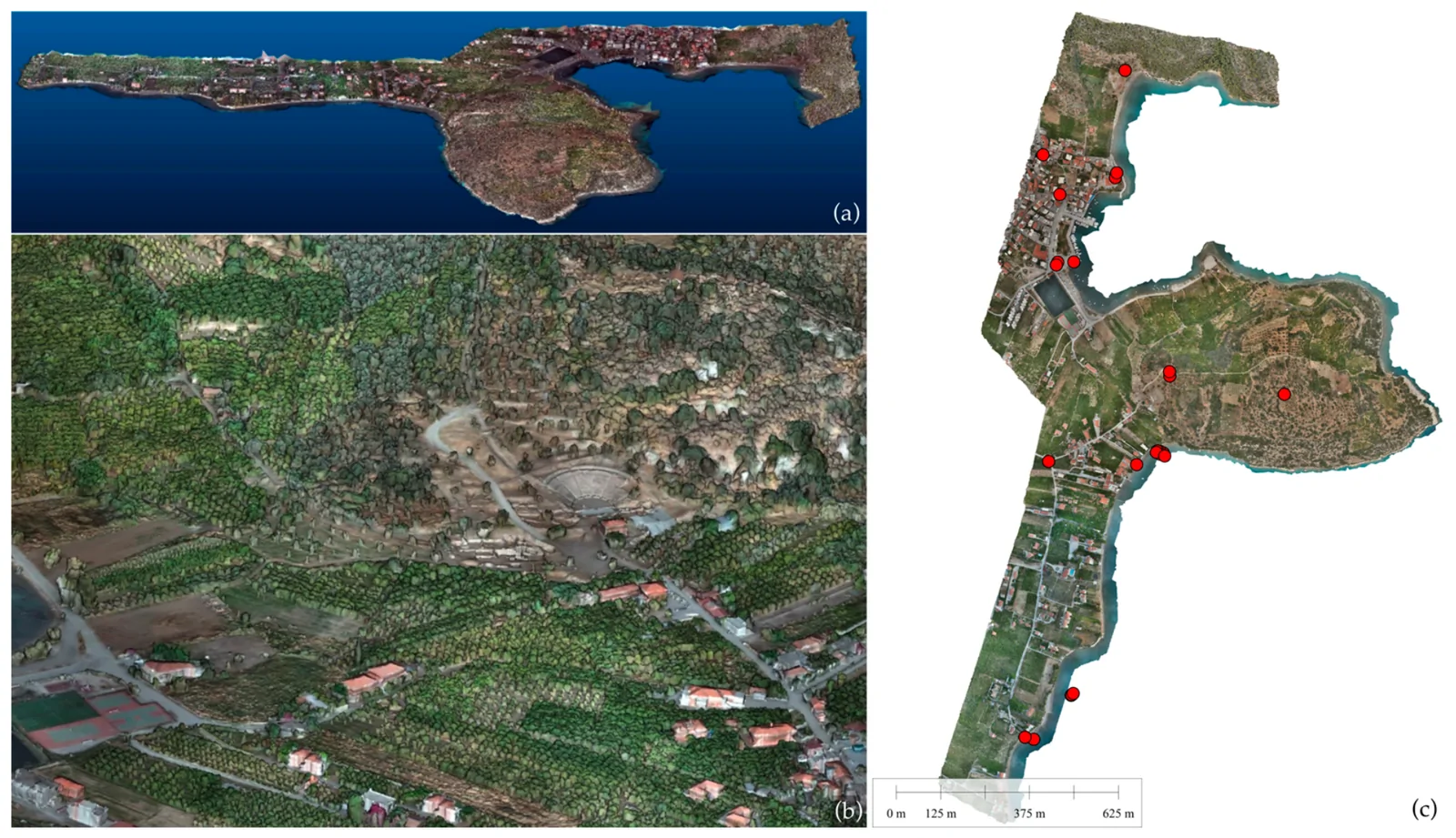
Geometric documentation of the Epidaurus site: (**a**) general view of the 3D model; (**b**) detailed view of the 3D model; (**c**) orthomosaic showing the spatial distribution of the GCPs (red circles).

The entire image dataset was processed in Agisoft Metashape Professional 2.1.2. The alignment of all photographs, constrained by a network of measured GCPs, produced a sparse point cloud of 8.6 million points with a GCP residual RMSE of 6.1 cm. No independent CPs were withheld for validation of the final UAV model. Further processing generated a dense 3D model consisting of approximately 150 million triangles. The final deliverables from this survey include a detailed 3D model and a high-resolution orthomosaic of the site, which was exported in tiles with a GSD of 15 mm, providing a critical tool for geospatial analysis and site management. Figure 18 presents general and detailed views of the 3D model together with the spatial distribution of the GCPs on the orthomosaic.

### 3.2. Underwater Documentation

Underwater surveys were carried out at three pilot sites, namely, Epidaurus in Greece, Roseninsel in Germany and Argilliez in Switzerland. We employed underwater photogrammetry, flash LiDAR mounted on a USV, and, where applicable, complementary acoustic sounding (single-/multibeam echosounders). The resulting photogrammetric, acoustic and flash LiDAR datasets were acquired as independent documentation products addressing different spatial extents, archaeological targets and environmental conditions. In the present study, these modalities are considered complementary in an operational and information sense; they were not systematically co-registered, fused or quantitatively compared to establish geometric agreement between sensors.

The flash LiDAR employed at the three underwater pilot sites was CSEM’s Airswim direct time-of-flight system, using 532 nm active illumination and a 128 × 128-pixel array of single-photon avalanche diodes. The system provides an adjustable underwater field of view (FOV) of 5–18° and an underwater measurement range of 0.7–15 m [64,112]. During the TRIQUETRA field campaigns, depth frames were acquired at 4 Hz, corresponding to a nominal point rate of approximately 65.5 × 10^3^ points s^−1^. Distance calibration was performed in air using a fixed-distance reference target, and underwater ranges were corrected using the refractive index of water (*n* = 1.333). The intrinsic equivalent in-water range precision is approximately 1.5 cm, derived from the calibrated in-air precision and converted to water using the same refractive-index correction. The LiDAR was mounted downward-facing beneath the USV, with navigation and orientation recorded using an SBG Systems Ekinox Micro inertial navigation system (INS; SBG Systems SAS, Carrières-sur-Seine, France) with two GNSS antennas on a 1.0 m baseline. Across all three campaigns, the horizontal and vertical positioning accuracies were 0.014 m, while the roll, pitch and heading accuracies were 0.57°. The lever-arm offsets between the LiDAR and INS were determined by direct physical measurement of the mounting geometry using a ruler; no dedicated boresight calibration was performed. INS trajectories were recorded in the native GNSS coordinate system, while the final LiDAR point clouds were transformed to a local relative coordinate system referenced to the first acquisition. System clocks were synchronized through a Network Time Protocol server on the INS. Depth frames were converted to 3D point clouds using inverse pinhole projection, followed by radius-based outlier removal, trajectory-based registration, statistical outlier filtering in CloudCompare and, where necessary, manual removal of residual noise and backscatter from suspended particles. Processing was performed using custom Python routines (Python 3.12.7) and CloudCompare 2.13.2 [113].

#### 3.2.1. Epidaurus

**Figure 19:**
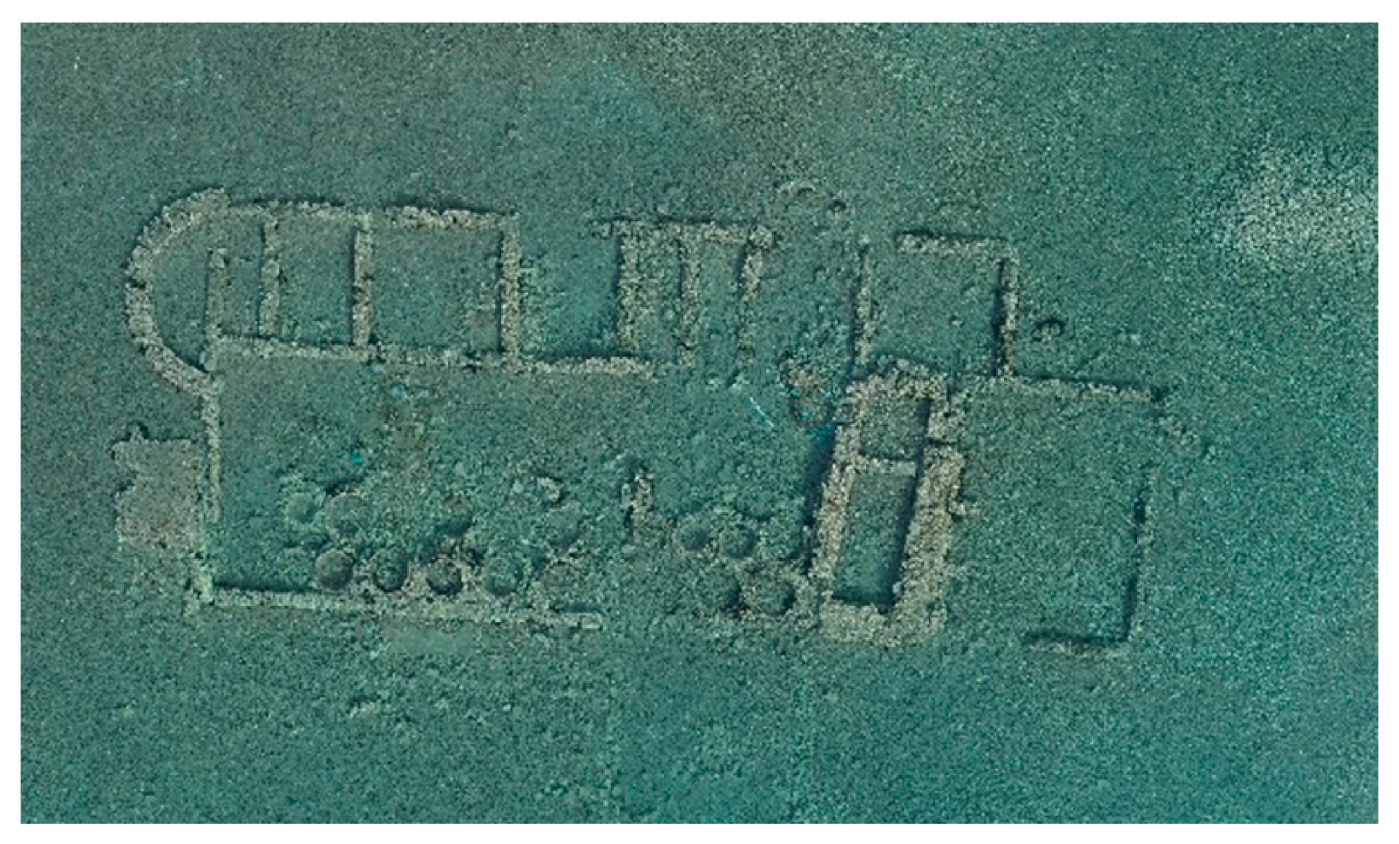
Aerial view of the Roman Mansion.

The underwater photogrammetric documentation focused on the submerged Roman “Mansion” (Figure 19) in the south bay of Epidaurus, a site that demanded a specialized approach due to significant operational challenges. The work was built upon established methodologies for underwater 3D reconstruction, which emphasize the critical importance of careful image collection and processing [31]. The survey was complicated by the extremely shallow depths in many areas (less than 0.5 m), which precluded the use of standard diver-operated camera systems, and the dense population of venomous “Diadema Setosum” sea urchins on the ruins, which posed a considerable safety risk.

To overcome these issues, an innovative data capture method was devised. Two GoPro HERO11 Black cameras were mounted 1.2 metres apart on a rigid rod, which was then fixed beneath a diver’s buoy. A diver could then safely navigate this rig from the surface, capturing 13,529 images in timelapse mode to ensure systematic and dense coverage. For capturing finer details in deeper, more accessible sections, an additional 308 images were taken with a medium-format Hasselblad X1D II camera (Victor Hasselblad AB, Gothenburg, Sweden) in a waterproof housing. To support metric georeferencing, a network of 24 control points was measured, with some placed in shallow waters, and scale bars were used in the deeper areas. No independent CPs were withheld for validation of the final underwater photogrammetric model.

**Figure 20:**
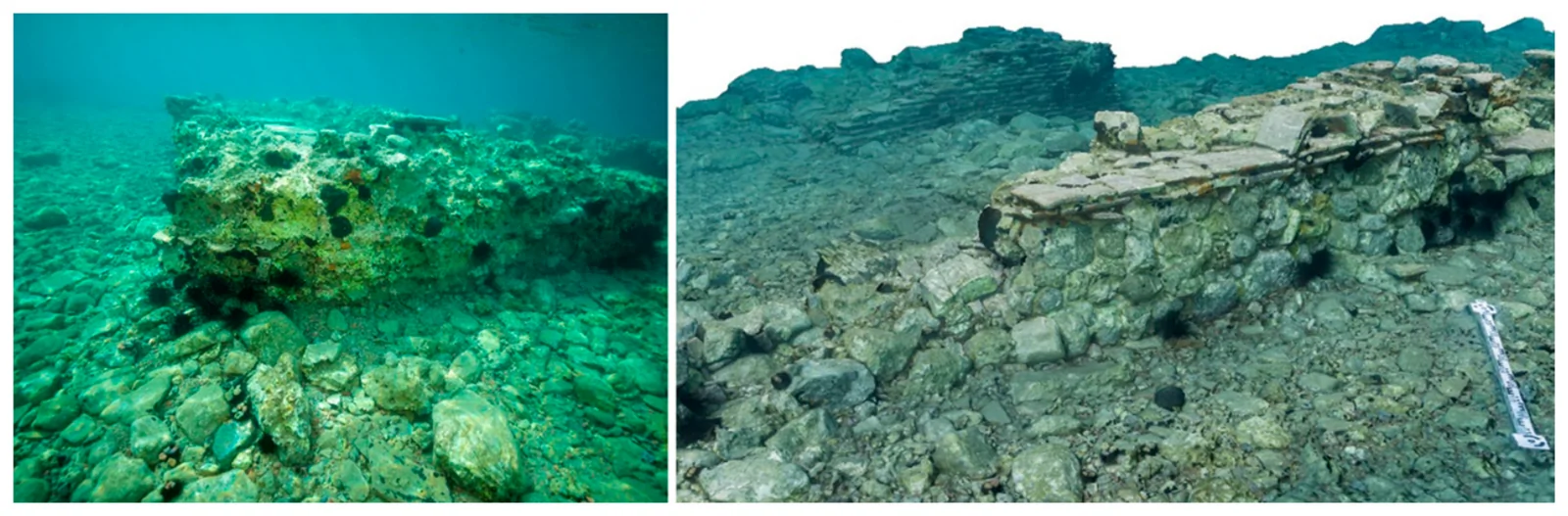
Photograph (**left**) and snapshot of the 3D model (**right**) of the Roman Mansion.

This multi-camera dataset was processed in Agisoft Metashape Professional 2.1.2, demonstrating the effectiveness of underwater photogrammetry for creating high-resolution models of submerged sites [31]. The images were aligned to produce a sparse point cloud of 11.6 million points with an overall GCP residual RMSE of 5.2 cm. The final 3D model contains approximately 88 million triangles and the resulting orthomosaic of the underwater site has a GSD of 3 mm, enabling detailed structural assessment of the antiquities (Figure 20).

In May 2024, a bathymetric survey of the north and south bays was conducted using an R2Sonic 2020 multibeam echosounder (MBES; R2Sonic, LLC, Austin, TX, USA), operated at 700 kHz with a 90° swath, together with an integrated Valeport sound velocity sensor (Teledyne Valeport Ltd., Totnes, UK), an Applanix SurfMaster inertial measurement unit (IMU) and dual Trimble GNSS receivers. Real-time sound velocity was continuously measured at the transducer head and used for beam steering and beamforming. Sound-velocity-profile corrections through the water column were not applied because of the very shallow depths within the survey area. The system was installed on a small polyethylene survey boat and received RTK corrections through the MetricaNET NTRIP service. Water-level variations were compensated in real time using RTK-tide. Vertical coordinates were referenced using the EGM2008 geoid model. Prior to mobilization, the physical lever-arm offsets between the primary GNSS antenna, the IMU and the acoustic centre of the MBES transducer were measured in the vessel reference frame using a FARO Focus S150 laser scanner (FARO Technologies, Inc., Lake Mary, FL, USA). Before each survey use, these offsets were refined by a few millimetres using the GNSS Azimuth Measurement Subsystem (GAMS) calibration procedure in MV-POSView 11.20. Patch-test calibration was subsequently performed in AutoPatch to determine and correct residual roll, pitch, yaw and latency misalignments. Owing to the draught of the MBES transducer mounting, the system could not operate in water shallower than approximately 2 m; the survey therefore targeted the deeper parts of the north and south bays. The survey was conducted at an average vessel speed of approximately 3.0–3.5 knots (1.5–1.8 m s^−1^). Survey-line spacing was dynamically adjusted to provide approximately 50–70% swath overlap, ensuring gap-free coverage under the shallow and spatially variable depth conditions. Approximately 81 million points were acquired in the south bay and 81.5 million points in the north bay, corresponding to an average density of approximately 700 points m^−2^. MBES data acquisition and processing were performed using the Beamworx software suite. Raw measurements were recorded in NAVAQ, while Autoclean was used for denoising, outlier rejection and removal of unnecessary overlapping measurements. Survey trajectories were edited and post-processed using TrajectEdit 2023.2. The processed bathymetric data were exported as an LAZ point cloud and as a GeoTIFF DTM with a 0.5 m sampling distance.

**Figure 21:**
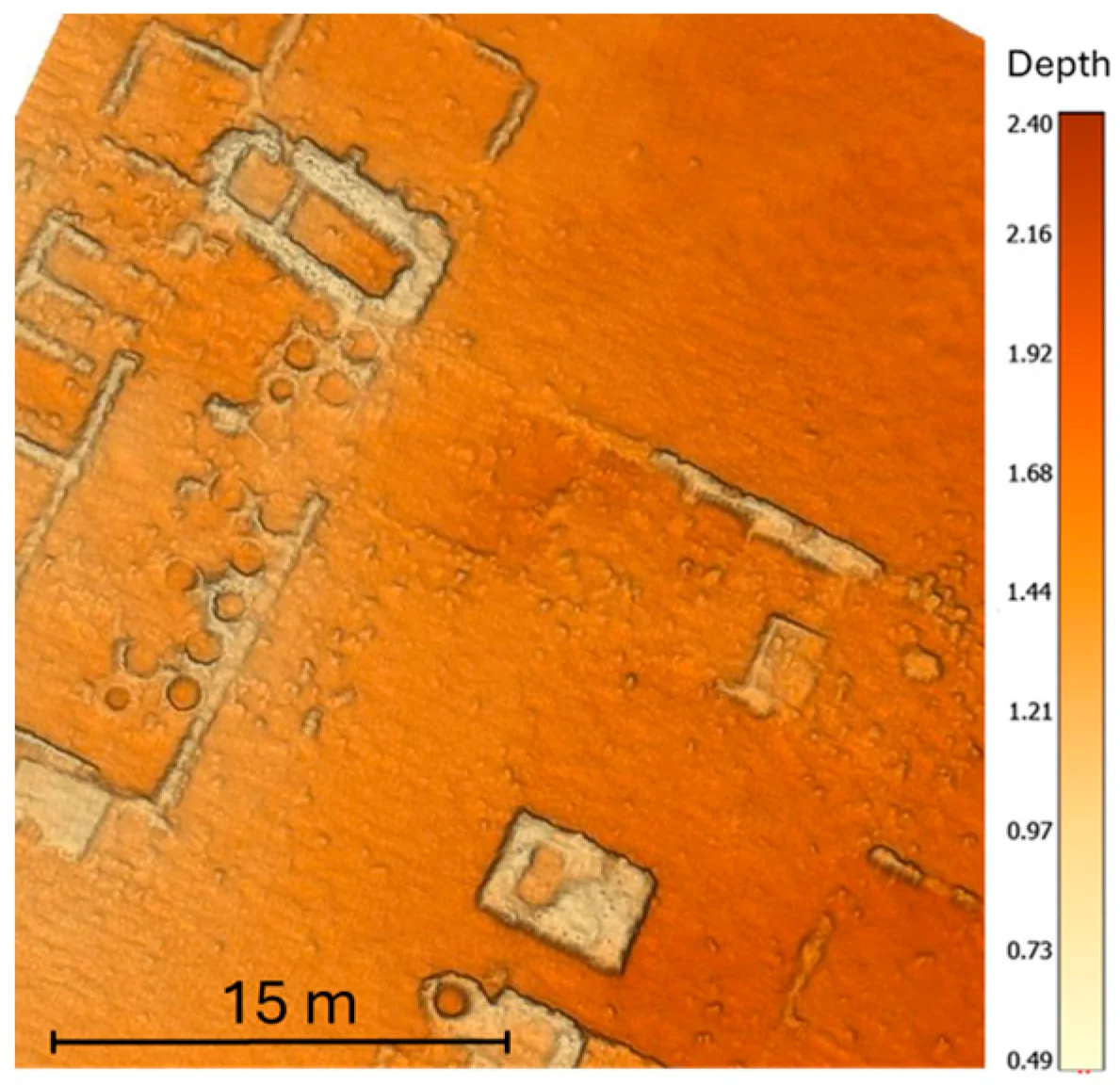
Point cloud section of the submerged Roman villa at Epidaurus, obtained from the flash LiDAR survey. The point cloud is visualised in an orthogonal projection using CloudCompare.

Furthermore, the Airswim flash LiDAR was deployed at Epidaurus in May 2025 on a BathyDrone Sumo USV to document the submerged remains of the Roman villa. The survey was conducted over two consecutive days, with approximately 160 raster-pattern transects completed in about four hours each day. On the second day, the transects were oriented perpendicular to those of the first day to improve coverage of the extremely shallow site. An 18° FOV was used, resulting in a footprint of approximately 16 cm at 0.5 m depth and 32 cm at 1 m depth. Satellite-derived Secchi-depth estimates exceeded 10 m near the survey area, while weather conditions ranged from sunny to overcast with occasional moderate rain. The two datasets covered an area of approximately 52 × 46 m and were combined into a single point cloud, achieving 98.6% completeness within the double-covered area using a 5 cm evaluation grid. No independent control or reference measurements were available for validation of the flash LiDAR dataset. The flash LiDAR dataset is considered here as an independent active-ranging record and was not used for quantitative surface-to-surface comparison with the photogrammetric model. The resulting point cloud is shown in Figure 21.

#### 3.2.2. Roseninsel

For the UNESCO World Heritage property at Roseninsel, there existed neither a bathymetric map nor a 3D documentation of the submerged remains of the prehistoric pile dwellings which can be found in the shallow waters around the island. To address these documentation gaps, dedicated sonar and underwater-photogrammetric campaigns were conducted using DLR’s USVs, followed by a flash LiDAR campaign targeting selected submerged archaeological remains.

**Figure 22:**
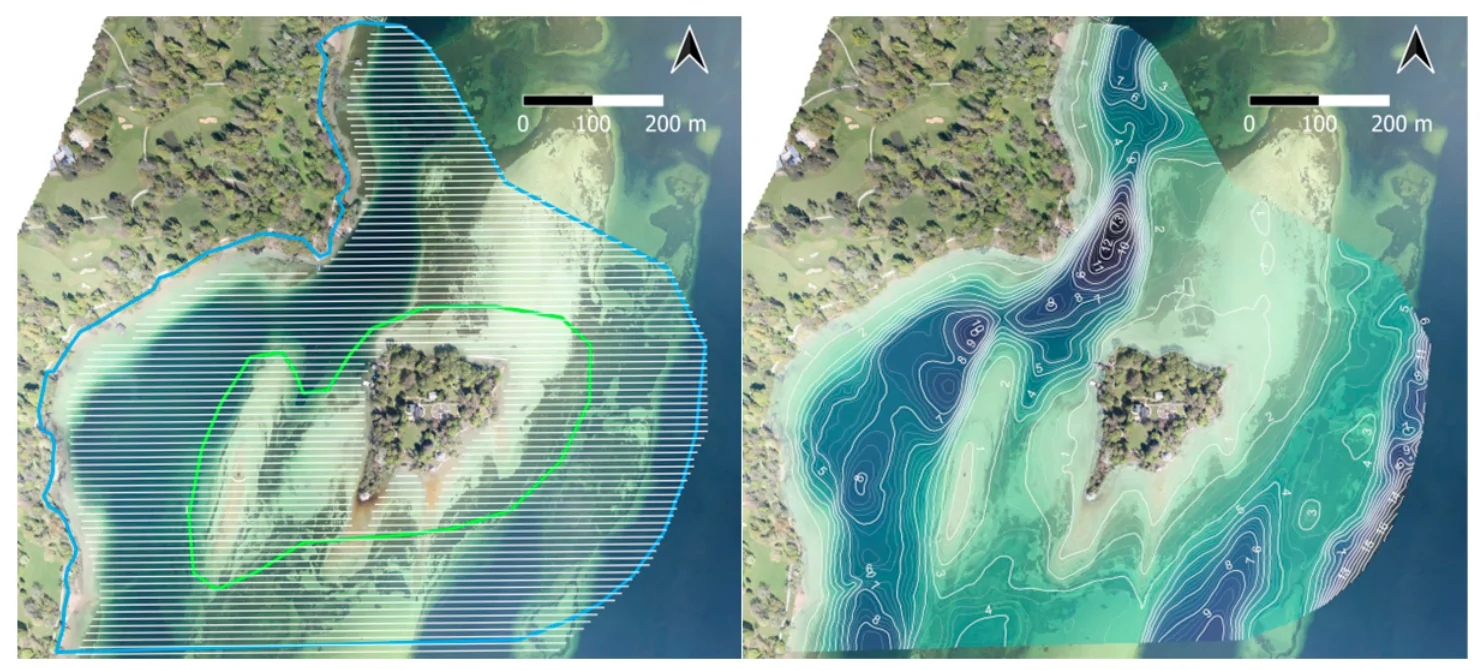
(**Left**): Core zone (green) and buffer zone (blue) of the UNESCO World Heritage Property at Roseninsel. Sonar survey tracks are depicted in white. (**Right**): Resulting bathymetric map.

The first campaign was conducted in July–August 2023 using DLR’s ultra-compact “La Plancha” USV (approximately 54 cm long), equipped with a Blue Robotics Ping single-beam echosounder operating at 115 kHz and RTK GNSS. The system was used to collect bathymetric information over the UNESCO World Heritage property, including both the core and buffer zones (Figure 22, left). The USV autonomously collected data along approximately 60 km of east–west survey lines with a spacing of 10 m, navigating at approximately 0.8 m/s and recording depth measurements at 10 Hz, corresponding to an along-track measurement spacing of approximately 8 cm. In the shallowest areas and close to the shoreline, the USV was operated by remote control to avoid collisions or grounding. Measured water depths ranged from approximately 0.32 to 14 m.

Because the campaign extended over several survey days, variations in lake level were monitored and subsequently corrected during processing. Five consecutive sonar measurements were averaged, resulting in an effective along-track sampling interval of approximately 0.4 m, and remaining outliers were removed manually in ReefMaster 2.0 [114]. Depth values were additionally corrected for the approximately 4 cm vertical offset between the water surface and the sonar transducer and normalized for day-to-day water-level variations using the water level measured by RTK GNSS during the first survey day as the reference. Comparison with reference measurements also revealed a small scale factor and offset in the sonar depths, which were applied to the raw measurements according to *depth_corr_* = 0.98 *depth_raw_* − 0.06 m. The processed measurements were interpolated to derive a bathymetric raster with a 1 m × 1 m cell size (Figure 22, right). The reference measurements comprised observations from a second echosounder and tape measurements at 62 locations independent of the survey points and covering the surveyed depth range. The resulting vertical RMSE was 10 cm relative to the tape-measure observations.

In a second campaign, carried out in summer 2024, a new, photogrammetric method for the documentation of the pile dwelling remains in the shallow waters around Roseninsel was tested. With a downward looking camera, mounted a few centimetres below the waterline on DLR’s multi-purpose USV LimnoVIS, a triangle-shaped area of approximately 1000 m^2^ was photographed. This area at the northeast tip of Roseninsel contains the highest density of remains (wooden timbers and poles) of the Celtic pile dwellings at the site and was lying at a depth of approximately 1 m during the campaign. About 10,000 nadir-oriented, overlapping images were collected over the area. RTK GNSS location and vehicle orientation were also recorded for each image.

**Figure 23:**
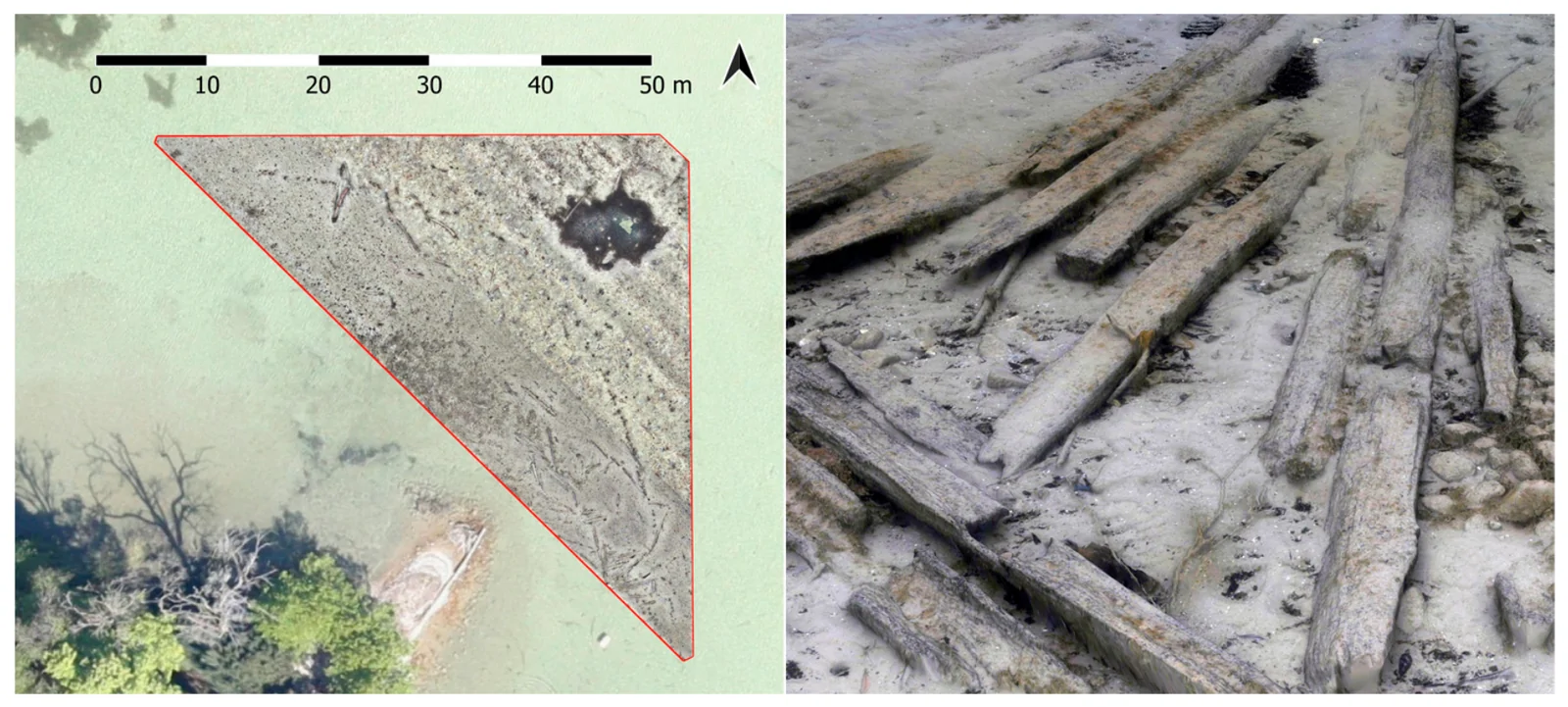
(**Left**): Orthomosaic of the area with the highest density of pile dwelling remains at the northeastern tip of Roseninsel. (**Right**): Image detail of the generated high-resolution 3D model.

The images were preprocessed by correcting and matching their brightness and colour. The main processing was conducted using Agisoft Metashape Professional 2.1.1. From the overlapping images and the recorded position and orientation data, a 3D point cloud and depth maps were calculated. Three wooden erosion markers with known coordinates, installed in 2014 as part of a 25 × 25 m monitoring grid, were initially used as independent reference points to assess a preliminary model generated solely from the RTK GNSS camera positions and INS orientations; the maximum positional deviation at these markers was below 10 cm. The same three markers were subsequently introduced as GCPs in the final adjustment. As no additional independent CPs were available, the positional accuracy of the final model could not be independently validated. From the point cloud and the depth maps, a 3D model and an orthomosaic with a GSD of approximately 1 mm were derived (Figure 23).

**Figure 24:**
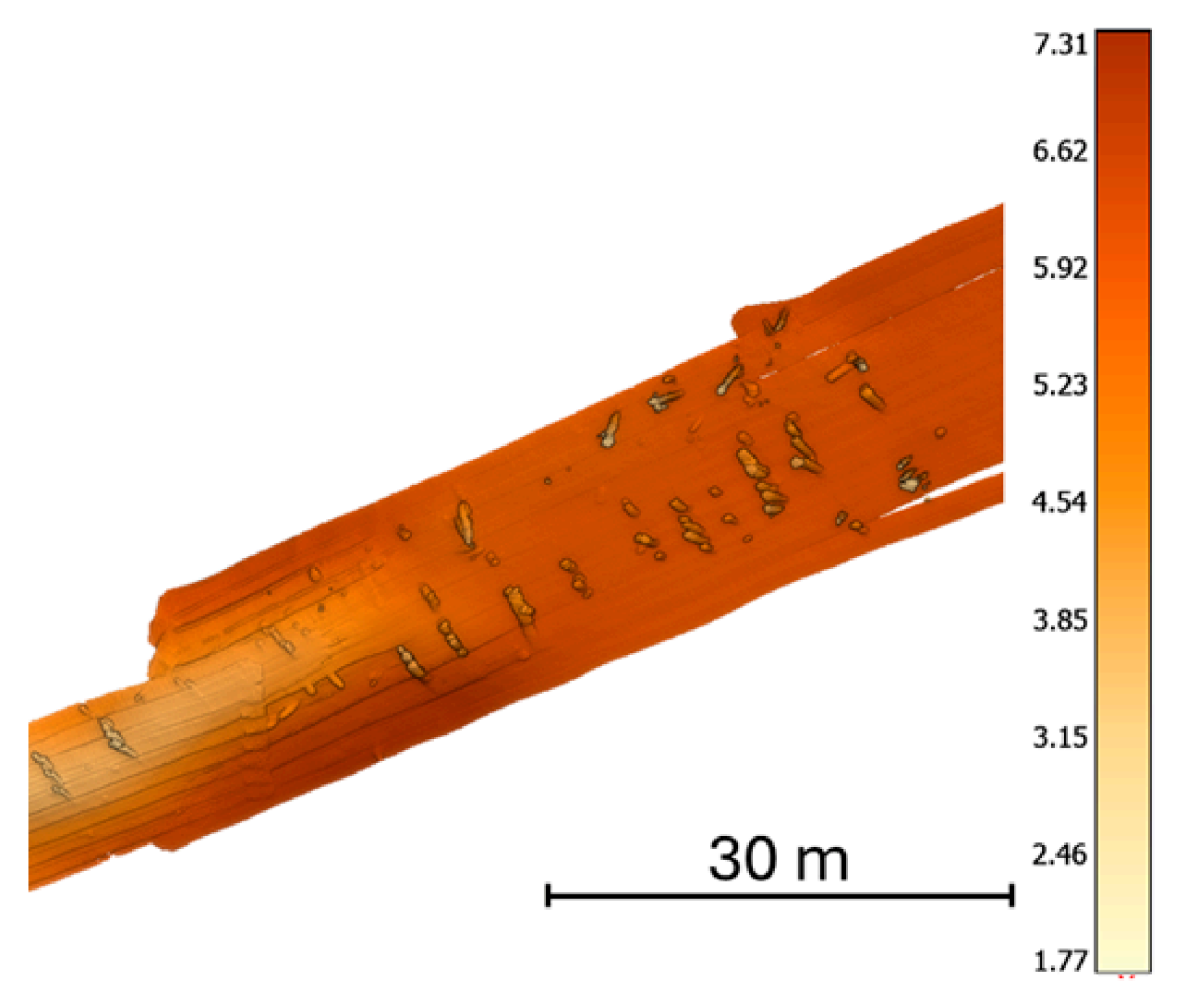
Point cloud section of the wooden remains of the medieval bridge at Roseninsel obtained from the flash LiDAR survey. The point cloud captures the distribution and orientation of surviving structural elements on the lakebed and is shown in orthogonal projection. The point cloud is visualised using CloudCompare.

In a third campaign, conducted in April 2025, the Airswim flash LiDAR was deployed on the LimnoVIS USV to document the remains of a medieval wooden bridge preserved below the lake surface. The same optical configuration as at Les Argilliez was used, corresponding to an FOV of approximately 15°, while the USV operated at approximately 0.5 m/s. Water conditions were relatively clear, with satellite-derived Secchi-depth estimates of approximately 5.2–6.5 m near the survey area, and LiDAR measurements reached depths of up to approximately 7.3 m. No independent reference targets were available during this campaign. A residual timing offset of approximately 0.3 s between the LiDAR timestamps and the INS trajectory was identified during processing, producing local misalignments between adjacent transects. The resulting point clouds nevertheless clearly recorded the preserved wooden elements on the lakebed, as illustrated in Figure 24.

#### 3.2.3. Les Argilliez

**Figure 25:**
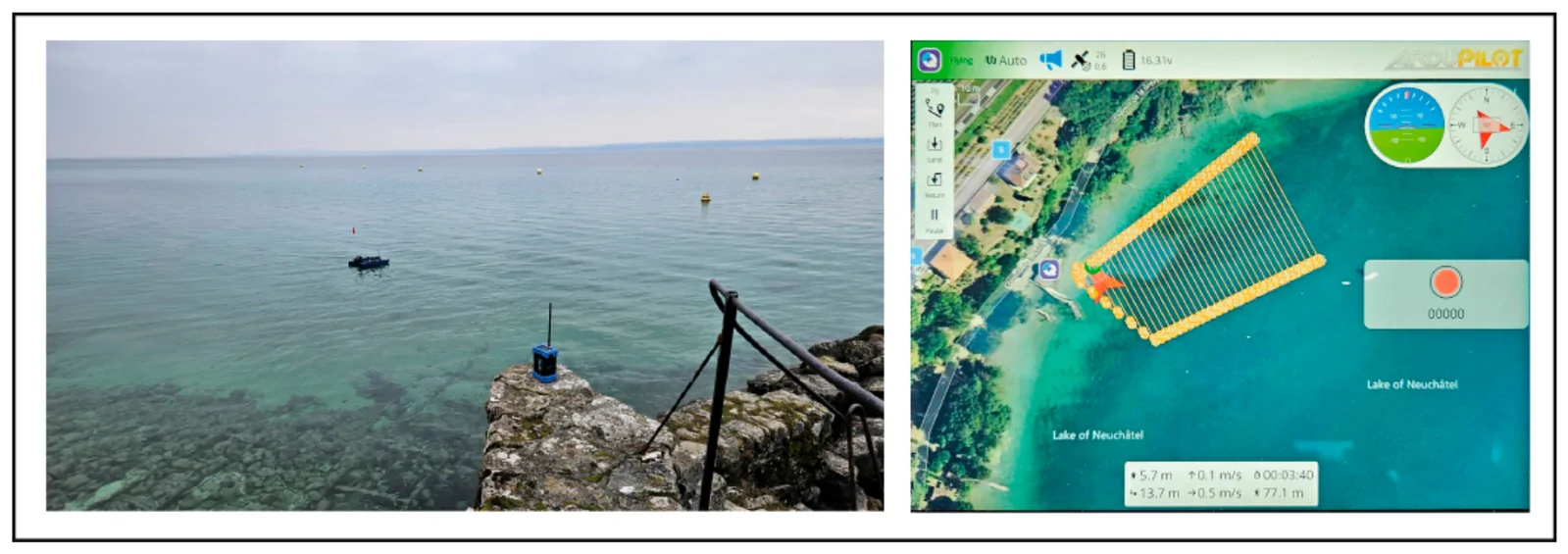
Image acquisition in Argilliez, using a USV that performs 2 m wide corridors.

An underwater photogrammetric campaign was carried out in early 2024 (January–April), before the appearance of vegetation to establish a site-wide geometric reference for future monitoring of erosion-front evolution and to document the current condition of the site. The surveys were carried out on the archaeological site of Gorgier—Les Argilliez using a BlueBoat remote-controlled USV (Blue Robotics Inc., Torrance, CA, USA) equipped with a GoPro HERO10 Black camera (GoPro, Inc., San Mateo, CA, USA) to generate a high-resolution 3D representation of the entire site. Primary image acquisition was carried out by following corridors every 2–3 m in width (Figure 25).

**Figure 26:**
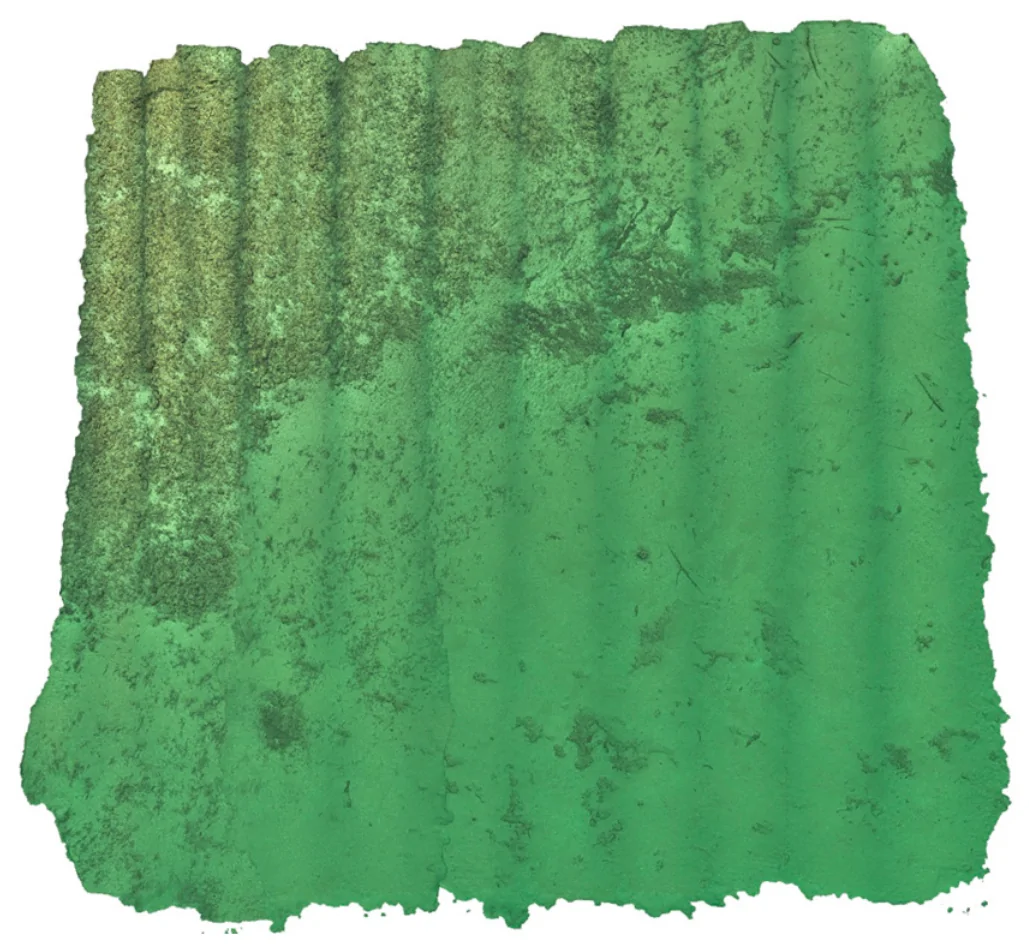
Three-dimensional model of Argilliez underwater cultural heritage site.

A total of 10,000 images was acquired and aligned for complete coverage of the area. The documented surface area represents approximately 15,000 m^2^ for a length of 200 m and a width of 75 m. The USV was guided by a Lowrance HDS 7 GPS system (Navico, Inc., Tulsa, OK, USA). Georeferencing and calibration of the model were carried out using 10 underwater GCPs measured with a Leica GNSS receiver adapted for underwater measurements. No independent CPs were withheld for validation of the final model. Photogrammetric processing was carried out using Agisoft Metashape Professional 2.2.2. The sparse point cloud has approximately 5.13 million points. Dense cloud generation and 3D mesh production followed (Figure 26). Texture mapping was then applied to the 3D mesh, using the oriented images.

**Figure 27:**
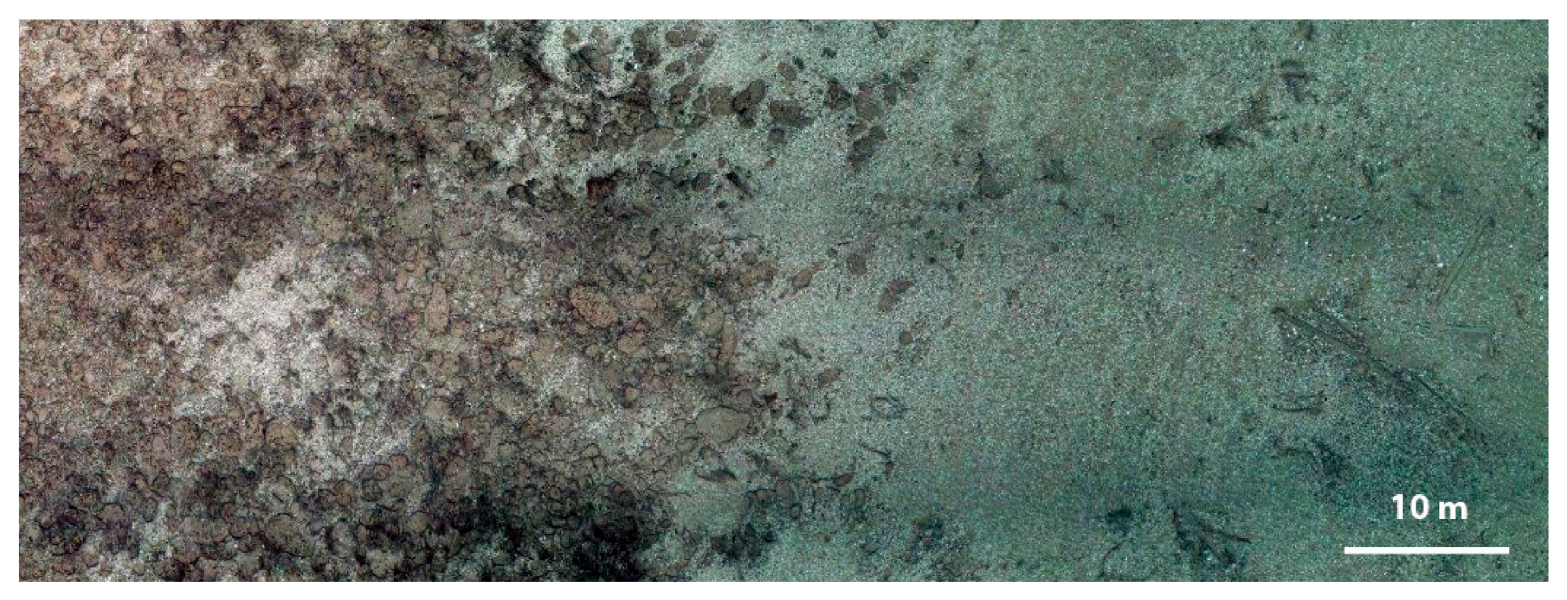
Detail of the orthomosaic of an area of the Neolithic village in Argilliez.

High-resolution orthomosaics were also generated (Figure 27). The main processing limitations were related to the size of the dataset and the available computing resources. The processing quality therefore had to be reduced, and the site was divided into two parts of 5160 m^2^ and 6300 m^2^ in order to handle the 3D representations.

**Figure 28:**
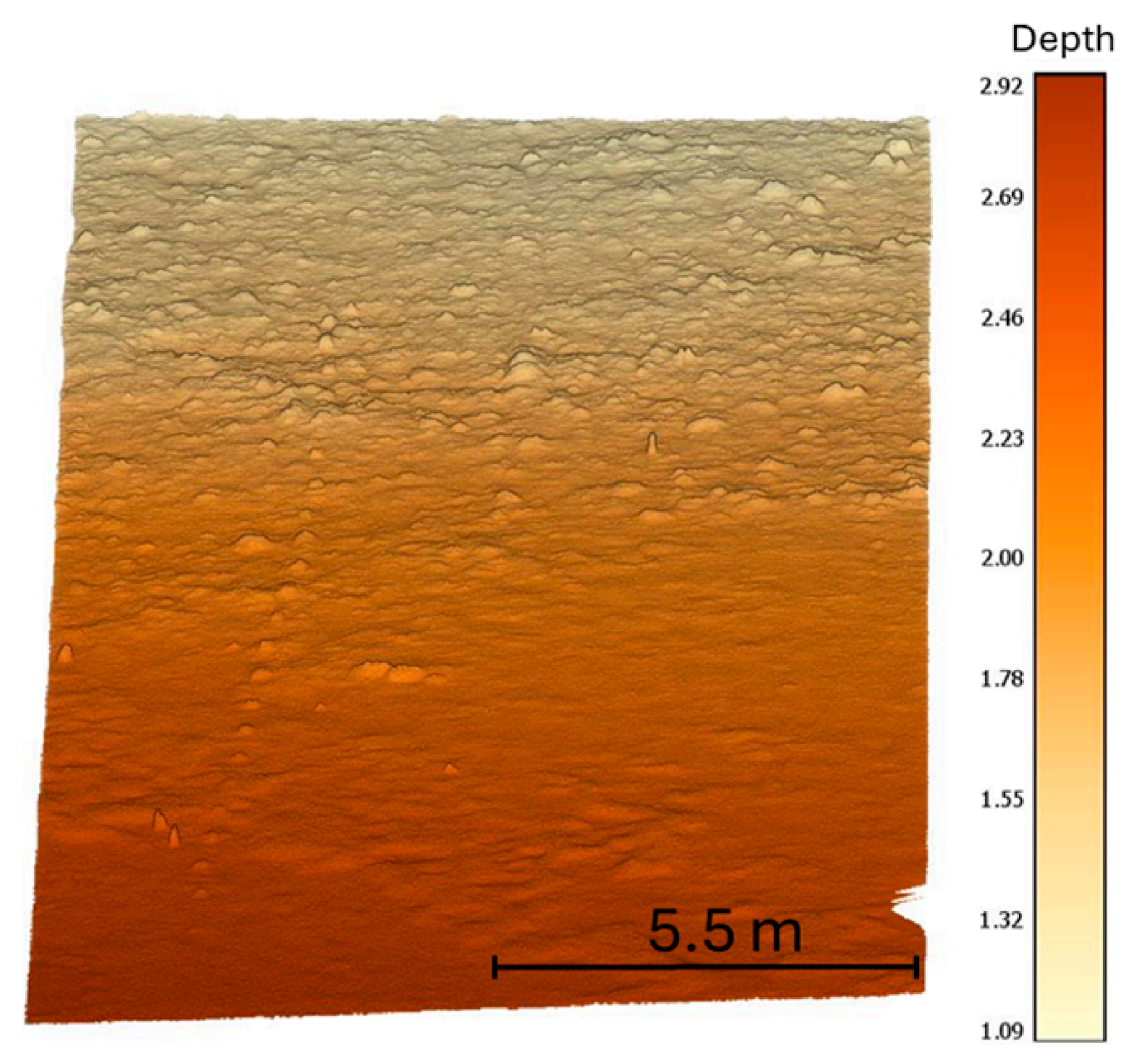
Point cloud section of Neolithic pile dwellings in Argilliez obtained from the flash LiDAR survey. The point cloud is visualised in an orthogonal projection using CloudCompare.

Furthermore, the Airswim flash LiDAR was deployed on a USV to document selected Neolithic pile-dwelling remains and to support repeated measurements along erosion-monitoring profiles. Four 30 m survey lines were established approximately perpendicular to the erosion front. Along one of these lines, terracotta bricks were installed as reference targets. An in-water intrinsic calibration was performed using a terracotta brick of known dimensions submerged at 2 m depth, resulting in an estimated focal length of approximately 31 mm and an FOV of approximately 15°. Repeatability was evaluated by surveying the same profile at USV speeds of 0.5, 0.7 and 1.0 m/s; the maximum segment-wise depth deviation was 3.835 cm, compared with an intrinsic equivalent in-water range precision of approximately 1.5 cm. The four survey lines were also surveyed simultaneously with a single-beam echo sounder (SBES), with maximum LiDAR–SBES depth differences of approximately 20 cm. These differences include the effects of relative sensor mounting, sensor offset and residual range-calibration uncertainty and therefore do not represent the intrinsic LiDAR precision. Water clarity near the surveyed area corresponded to a satellite-derived Secchi-depth estimate of approximately 6.4 m. The resulting point clouds provide a high-resolution geometric record of the submerged archaeological features and erosion-monitoring profiles, as shown in Figure 28.

## 4. Results

### 4.1. Geometric Documentation Outputs Across the Pilot Sites

The documentation campaigns carried out across the pilot sites resulted in a broad set of geospatial 2D, 2.5D and 3D products covering inland, coastal and shallow-water cultural heritage environments, including both lacustrine and shallow-marine contexts. These products include high-resolution orthomosaics, DSMs/DTMs, dense point clouds, textured 3D meshes, bathymetric datasets and underwater point clouds derived from optical, acoustic and active ranging sensors. Rather than being considered as isolated visual products, these outputs form a set of site-specific geometric evidence layers that describe the current spatial condition of each heritage asset and its surrounding environment.

Across the eight pilot sites, the relationship between conservation need and geometric documentation can be grouped into four recurring documentation roles. First, high-detail archaeological recording was required where the primary objective was the metric representation of exposed or submerged architectural remains, as in Kalapodi, Aegina and selected areas of Epidaurus. Second, at Ventotene, Choirokoitia and parts of Aegina and Epidaurus, the documentation needed to extend beyond the heritage remains themselves to include the surrounding cliff, slope or coastal morphology, making spatial coverage and elevation representation particularly important. Third, Smuszewo represents the clearest multi-temporal case, where repeated UAV surveys were used to record lake-margin, vegetation and surface changes through time rather than to maximize local geometric detail. Finally, at Epidaurus, Roseninsel and Argilliez, underwater documentation requirements varied according to water depth, visibility, spatial extent and archaeological target, resulting in different information roles for photogrammetric, acoustic and active-ranging products. These recurring patterns show that the required geometric evidence differs according to the conservation problem and environmental setting, in terms of both spatial scale and the type of product needed.

The main result of the documentation work is therefore not a single 3D model, but a structured set of products with different interpretative roles. Orthomosaics provide planimetric evidence for mapping, visual inspection and comparison of surface conditions. DSMs and DTMs describe elevation, relief and terrain morphology. Textured 3D models combine geometric and visual information, supporting the interpretation of architectural remains and their spatial relationship with the surrounding environment. Underwater photogrammetric, acoustic and LiDAR-derived products extend this geometric record to submerged contexts, where direct observation and conventional mapping are often limited.

### 4.2. Land-Based Documentation Results

The land-based results reveal three distinct documentation roles: high-detail archaeological recording, documentation of heritage within its surrounding geomorphological context, and repeat-survey surface monitoring. Where the primary need is detailed recording of exposed archaeological remains, the generated 3D models and orthomosaics record the geometry and spatial arrangement of exposed structures, enabling the systematic visualisation of walls, foundations, excavation units and conservation-relevant surfaces. This is particularly relevant for sites where material deterioration, masonry instability or past restoration interventions require a detailed spatial record of the current condition.

At Kalapodi, the geometric products support the documentation of stone structures and the spatial organization of the sanctuary, providing a detailed baseline that can be related to environmental monitoring and material decay studies. At Aegina Kolonna, the 3D and 2D outputs capture both the archaeological remains and their coastal setting, including the spatial relationship between the excavated structures and the cliff edge. At the coastal area of Epidaurus, the documentation products cover a wider and topographically varied area, recording both the archaeological remains and their surrounding coastal terrain. These cases also show that the documentation roles are not mutually exclusive: at Aegina and coastal Epidaurus, high-detail archaeological recording is combined with broader terrain documentation because the adjacent coastal morphology is itself relevant to site vulnerability.

For sites affected by slope instability, erosion or steep relief, the results have an additional geomorphological value. In Ventotene, the generated products represent not only the archaeological remains of Villa Giulia, but also the surrounding promontory and cliff system. This allows the cultural heritage elements to be interpreted in relation to the coastal morphology that influences site vulnerability. In Choirokoitia, the elevation and surface products support the representation of the settlement within its steep topographic setting, where slope processes and rockfall hazards are relevant to site management.

At Smuszewo, the documentation products have a different role. The repeated UAV-derived products provide spatial evidence of seasonal and environmental changes in the lake-margin zone. The orthomosaics and surface models support the observation of water-level variation, vegetation change and surface indicators related to moisture conditions. In this case, the value of the geometric products lies less in the documentation of visible architecture and more in their capacity to record environmental proxies associated with the preservation state of buried wooden archaeological remains. Smuszewo therefore provides the clearest direct demonstration in this study of geometric documentation being used as multi-temporal evidence rather than solely as a baseline record.

Taken together, the terrestrial and coastal results show that the same broad product types—orthomosaics, elevation models, dense point clouds and textured meshes—do not have a uniform interpretative role across all sites. Their value depends on the heritage typology, the dominant hazard and the spatial scale of the conservation question. This confirms the need to interpret 3D documentation outputs as evidence layers tailored to specific documentation, monitoring and conservation needs, rather than as generic visualisation products.

### 4.3. Shallow-Water Documentation Results

The shallow-water documentation products extend the geometric record to submerged lacustrine and shallow-marine environments where cultural heritage remains are difficult to access, inspect or map using conventional field methods. Across Epidaurus, Roseninsel and Argilliez, three distinct information roles emerge from the shallow-water documentation: high-detail visual and geometric recording of submerged archaeological remains, broader characterization of underwater morphology and localized active-ranging documentation of selected submerged features.

At Epidaurus, the underwater products document the submerged Roman remains and the surrounding nearshore and bathymetric context. The optical photogrammetric model provides a detailed visual and geometric record of the shallow submerged masonry, while the flash LiDAR point clouds provide active-sensor representation of selected submerged structures. Considered at the site level, these independently acquired products illustrate different information roles: photogrammetry provides visually interpretable and detailed surface representations, whereas flash LiDAR provides active-ranging observations of selected submerged features. No quantitative geometric agreement between the two datasets is assessed in the present study.

At Roseninsel, the generated products address a different documentation problem: the mapping of submerged pile-dwelling remains and their surrounding lakebed context. The bathymetric and image-based outputs provide information at different spatial scales: the bathymetric product characterizes the broader shallow-water morphology around the island, whereas photogrammetry records a limited area containing a high density of archaeological remains at much finer detail. These products are therefore interpreted according to their distinct documentation roles rather than through direct geometric comparison.

At Argilliez, the documentation products provide a site-scale representation of a submerged prehistoric pile-dwelling landscape. The site-wide photogrammetric products provide broad visual and geometric documentation of the submerged archaeological landscape, whereas the flash LiDAR measurements provide localized active-ranging records of selected features. Their value is therefore interpreted in terms of different documentation roles and spatial coverage rather than geometric agreement between the datasets. For the flash LiDAR measurements at Les Argilliez, repeated acquisition of the same survey profile produced a maximum segment-wise depth deviation of approximately 3.8 cm, defining the measurement repeatability under the tested conditions. Independent comparison with simultaneously acquired SBES measurements along the four monitoring lines showed maximum depth differences of approximately 20 cm; these differences characterize the observed inter-sensor depth agreement and are distinct from the intrinsic equivalent in-water range precision of approximately 1.5 cm.

Across the underwater sites, the results indicate that the different sensor-derived products address different documentation needs rather than forming geometrically fused multi-sensor datasets. Optical models provide visual interpretability and fine surface detail where water clarity permits, bathymetric data provide broader underwater terrain context, and flash LiDAR point clouds provide active-ranging measurements of selected submerged features. Their complementarity in the present study is therefore operational and information-based, reflecting differences in spatial coverage, target type, environmental applicability and information content. No systematic co-registration, surface-difference analysis or inter-sensor agreement assessment was performed.

### 4.4. Monitoring-Relevant Information Derived from the Documentation Products

The generated products provide several types of monitoring-relevant information, although the level of monitoring demonstrated in the present study differs among the pilot sites. First, for most sites, the surveys establish georeferenced baseline datasets that can support future repeat acquisition and change assessment, provided that subsequent surveys are acquired within a consistent spatial reference and with comparable coverage and resolution. Smuszewo represents the clearest multi-temporal case in the present study, because repeated UAV surveys already enable the direct observation of variations in lake-margin and surface conditions through time.

Second, the products support the spatial localization and geometric characterization of features relevant to site vulnerability. These spatial indicators provide geometric inputs that can contribute to risk assessment when considered together with the relevant hazard, environmental, material and condition information. In terrestrial and coastal sites, these include exposed masonry, cliff edges, steep slopes, excavation boundaries, terrain discontinuities and areas where architectural remains are directly adjacent to unstable geomorphological settings. In lacustrine and underwater sites, they include submerged structures, sediment-covered zones, erosion fronts, shallow-water margins and areas where organic remains may be affected by changing hydrological or biological conditions.

Third, the documentation products provide spatial reference layers through which heterogeneous observations can be spatially associated at the site level. Environmental monitoring data, material analyses, geophysical measurements, field observations and heritage management information can be spatially associated with the relevant 2D, 2.5D and 3D products. The georeferenced products can therefore serve as potential spatial inputs to digital twin or decision support environments; their functional integration and use within such environments are not evaluated in the present study.

The monitoring and conservation relevance of the different product types is summarized in Table 3. The table does not classify the sensors themselves, but the type of information extracted from the resulting products. This distinction is important because the same sensor-derived product may support different heritage management tasks depending on the site context.

Overall, the results distinguish between geometric documentation used directly for multi-temporal observation and datasets that primarily establish a baseline for future monitoring. Smuszewo demonstrates the former through repeated UAV acquisitions, whereas the products generated at the other pilot sites principally provide reference datasets that can support subsequent repeat surveys and condition comparison. Across both cases, their value lies in transforming visible, submerged and terrain-related site conditions into measurable and reusable spatial evidence whose monitoring relevance depends on the conservation question, spatial scale and the geometric consistency and repeatability achievable between successive surveys.

## 5. Discussion

The results of this study show that multi-sensor geometric documentation can provide more than visual records of cultural heritage sites. Across the eight pilot sites of the TRIQUETRA project, the generated products act as spatial evidence layers that describe the present condition of heritage assets and their surrounding environments in a measurable form. This is particularly relevant for sites exposed to environmental, climatic, geological or hydrological processes, where documentation must support not only representation, but also interpretation, comparison and future conservation planning.

The main contribution of the study is the integrated interpretation of how geometric documentation requirements and the information value of the resulting products vary with the conservation problem and environmental setting. The work does not propose a single universal method, nor does it aim to compare sensors under controlled benchmark conditions. Instead, the results distinguish different documentation roles according to the spatial evidence required: high-detail recording of archaeological remains, representation of heritage together with surrounding terrain or coastal morphology, repeat-survey observation of environmental surface change, and submerged-site documentation at different spatial scales. This distinction is important, because the value of a documentation product depends on the conservation question it supports. An orthomosaic, a DSM, a textured mesh, a bathymetric model or an underwater point cloud does not have the same role in all sites. In one case, it may support the mapping of exposed architectural remains; in another, it may describe the relation between heritage structures and unstable terrain; in another, it may document submerged features that are difficult to inspect directly.

This interpretation shifts the emphasis from product generation to information value. High geometric detail is important, but it is not the only criterion for assessing the usefulness of the results. A product is valuable when it captures the spatial condition that is relevant to a specific monitoring, risk or conservation need. For most pilot sites, the generated datasets primarily establish site-specific geometric baselines for subsequent repeat surveys, whereas at Smuszewo the repeated acquisitions already demonstrate their use for multi-temporal observation. Their common feature is therefore not identical resolution, density or coverage, but their capacity to provide spatial evidence at a scale and level of detail appropriate to the conservation problem being addressed.

A further contribution of the study is the comparative interpretation of the different information roles of optical, acoustic and active-ranging approaches in shallow-water heritage documentation. Optical photogrammetry provides visually interpretable and geometrically detailed surface representations where visibility, texture and access conditions are favourable; acoustic sounding extends the documentation to broader underwater terrain and bathymetric context; and flash LiDAR provides localized active-ranging observations of selected submerged features. Because these modalities were acquired independently and, in several cases, addressed different spatial extents or archaeological targets, no systematic co-registration, fusion or quantitative inter-sensor agreement assessment was undertaken. Their complementarity in the present study is therefore demonstrated at the level of documentation role and information content: together, the different approaches extend the types and spatial scales of evidence that can be obtained under different underwater site and environmental conditions.

The Epidaurus MBES survey provides a concrete example of these modality-specific advantages and limitations. Its principal advantage was the efficient acquisition of dense, spatially continuous bathymetric information over the deeper parts of the north and south bays, where approximately 162.5 million soundings were collected at an average density of ~700 points m^−2^ and used to generate a 0.5 m bathymetric DTM. This broader seabed representation complements the much more localized, high-detail recording provided by underwater photogrammetry and flash LiDAR and is particularly suited to interpreting harbour and seabed morphology. More generally, acoustic measurements are independent of ambient illumination and optical water visibility, making MBES suitable for broader underwater mapping where optical methods may be constrained. However, MBES provides lower lateral detail than close-range underwater photogrammetry for small, thin or low-relief archaeological features, while the quality of the resulting bathymetric model remains dependent on acquisition geometry, platform motion and sensor integration. In the present Epidaurus campaign, a more immediate operational limitation was imposed by the draught of the transducer mounting, which prevented MBES acquisition in water shallower than approximately 2 m. Consequently, the ultra-shallow Roman Mansion could not be mapped with the MBES configuration and was instead documented using underwater photogrammetry and flash LiDAR. These characteristics indicate that MBES is particularly advantageous where broad and spatially continuous bathymetric context is required and sufficient water depth permits vessel operation, whereas complementary optical or active-ranging methods may be preferable for ultra-shallow areas and for the detailed recording of small archaeological features. More broadly, these modality-specific trade-offs reinforce the fit-for-purpose interpretation adopted in this study: the value of each geometric product depends not only on its technical characteristics, but also on the conservation question it is intended to inform.

The results also clarify the specific role of geometric documentation in supporting risk-informed conservation. The products generated here are not risk maps and do not, by themselves, quantify the probability or severity of future damage. Their value lies in providing the spatial framework within which risk-relevant features can be located and interpreted. Exposed masonry, cliff edges, slopes, erosion fronts, sediment-covered areas, lake margins and submerged structures can be represented in relation to their surrounding environment. More specifically, at cliff-dominated sites, high-resolution photogrammetry can support the analysis of landslide processes by documenting predisposing conditions related to cliff geometry, geological layering as well as rock mass discontinuities and joints. These discontinuities help define potentially unstable rock blocks geometries and volumes and therefore support the assessment of their proneness to failure. In this context, photogrammetric information can contribute to landslide susceptibility mapping by providing spatial evidence of the predisposing conditions that control the spatial likelihood of landslide occurrence. However, geometric documentation alone is not sufficient to define landslide-hazard scenarios, which additionally require information on preparatory factors and triggering actions. Based on these considerations, such spatial documentation provides geometric evidence that can be considered together with hazard, environmental, material and condition information when assessing site vulnerability and defining conservation priorities.

The generated products also provide a basis for future monitoring, condition assessment and structural inspection, applications for which UAV-photogrammetric 3D models have also been successfully employed in recent heritage studies [115]. For most pilot sites, however, the present products should be regarded as baseline datasets rather than completed monitoring series; Smuszewo is the clearest exception, where repeated UAV acquisitions already enable multi-temporal observation of environmental surface conditions. Robust quantitative change assessment would require repeatable acquisition geometry and spatial coverage, stable georeferencing within a common reference frame, reliable co-registration of datasets acquired at different epochs, and a level of geometric uncertainty sufficiently low relative to the magnitude of the changes to be detected [43,45,46]. The present datasets therefore provide the initial spatial reference and geometric record from which such repeat-survey analyses can subsequently be developed.

A similar distinction applies to digital twin and decision support applications. The documentation products provide potential spatial inputs for such environments by representing heritage assets together with their physical context. They can act as spatial reference layers for linking environmental monitoring, material studies, field observations, geophysical information and conservation information [116,117]. Although the generated geometric products are accessible for visualisation through the TRIQUETRA Decision Support System (DSS) platform, the present study does not evaluate their functional integration with analytical or decision-support components, dynamic data updating, interoperability, or decision-support performance. Such integration requires additional data structuring, metadata management, interoperability, model simplification and visualisation procedures that are beyond the scope of the present study. A highly detailed mesh or dense point cloud may be useful for technical inspection, but may not necessarily be suitable for interactive use without appropriate processing and abstraction.

These interpretations must also be considered in light of the heterogeneity of the pilot sites, which is both a strength and a limitation of the study. It is a strength because it reflects real cultural heritage documentation conditions, where sites differ in accessibility, scale, material, exposure, visibility and conservation needs. It is also a limitation because the outputs cannot be directly compared as if they were produced within a controlled sensor-performance experiment. The study should therefore not be read as a ranking of technologies, but as an interpretation of how different sensor-derived products contribute to the documentation of complex heritage environments and provide different forms of spatial evidence. Accordingly, differences in resolution, point density and geometric accuracy should be interpreted within the acquisition and validation framework of each individual survey rather than used for direct performance comparisons across sites or sensing technologies.

Overall, the eight pilot sites show that the value of geometric documentation does not lie in uniformity of sensor, resolution or product type, but in the ability of the resulting spatial evidence to address the conservation problem at an appropriate scale and level of detail. The generated products consequently fulfil different roles, from detailed archaeological baseline recording and geomorphological contextualization to multi-temporal observation and modality-specific underwater documentation. Within the limitations identified above, these spatial records can support future condition assessment and repeat-survey comparison, contribute geometric evidence to risk-informed conservation, and provide potential inputs for digital twin and decision support applications.

## Figures and Tables

**Table 1:** Positioning of the present study relative to previous related TRIQUETRA publications. The table summarizes prior coverage and the specific contribution of the present study in terms of datasets, methodological reporting, results and integrated interpretation.

Pilot Site/Scope	Coverage in Previous TRIQUETRA Publications	Specific Contribution of the Present Study
Kalapodi	The geometric documentation and principal photogrammetric products of the sanctuary were previously presented as part of the documentation of the Greek TRIQUETRA pilot sites [51].	Provides a substantially more detailed and self-contained treatment of the complete geometric documentation campaign, from acquisition design and geodetic control to processing and high-resolution 2D/3D products, and interprets these products specifically in relation to the detailed recording and future condition assessment of exposed archaeological remains affected by environmental deterioration.
Ventotene and Santo Stefano	The Ventotene and Santo Stefano pilot site was previously addressed through high-resolution photogrammetric documentation, drone-based 3D modelling for engineering-geological analysis, and broader remote-sensing-based hazard investigations [54,55,56].	Provides a substantially more detailed and comprehensive geometric documentation account within the present study framework, covering both the archaeological remains and the surrounding coastal cliffs and reporting the complete set of 2D, 2.5D and 3D products. This enables the heritage asset as well as the geological and geomorphological setting controlling the vulnerability of the archaeological remains to be interpreted as a single spatial context.
Aegina	The geometric documentation of Aegina Kolonna was previously reported in the Greek TRIQUETRA pilot site study [51] and in UAV-focused heritage research [73], while the resulting dataset was also used in site-specific risk and conservation studies [52,53,57,58,59].	Provides a more complete and integrated presentation of the campaign, processing chain, accuracy assessment and full suite of geometric products, while interpreting the documentation not only as an archaeological record but also as spatial evidence of the relationship between exposed remains, excavation areas and the adjacent coastal terrain.
Choirokoitia	A previous TRIQUETRA-related study discussed monitoring and digital representation of the site [60], but did not present its complete geometric documentation campaign at the level of detail provided here.	Presents the detailed UAV-based geometric documentation of the pilot site and its resulting terrain and archaeological representations, establishing a high-resolution spatial baseline that captures both the settlement and its steep hillslope setting and provides a spatial reference for future repeat-survey assessment of morphological change and for the interpretation of rockfall-related vulnerability.
Smuszewo	UAV-based observations and environmental monitoring were previously presented in the context of climate-related risk identification and lakeshore monitoring [61,62].	Provides a substantially extended geometric and multi-temporal documentation dataset, including additional survey epochs and detailed photogrammetric products, and demonstrates their use for identifying lake-margin, water-level, vegetation and other surface changes through time. It therefore provides the clearest example in the study of geometric documentation progressing from baseline recording to repeat-survey evidence.
Epidaurus	Principal aerial and underwater documentation activities were previously introduced within the Greek TRIQUETRA pilot-site study [51], selected geometric products were subsequently used in coastal hazard-modelling applications [59], while flash LiDAR performance has been examined in sensor-focused research [64].	Provides the most complete and comprehensive geometric documentation account of the pilot site, encompassing terrestrial/coastal UAV photogrammetry, very-shallow-water photogrammetry, acoustic bathymetry and flash LiDAR. The study interprets the distinct information provided by these modalities across the coastal-to-submerged heritage setting rather than treating them as geometrically fused datasets.
Roseninsel	The bathymetric and shallow-water photogrammetric campaigns were previously reported in a dedicated site study [63], while flash LiDAR measurements were subsequently included in sensor-focused research [64].	Brings all geometric documentation modalities applied at the pilot site into a common, detailed account, encompassing bathymetric, photogrammetric and active-ranging products and clarifying their different information roles: broad lakebed morphology versus high-detail recording of submerged archaeological features.
Les Argilliez	Previous TRIQUETRA publications have focused primarily on the development and application of underwater flash LiDAR [64,65].	Introduces the site-wide underwater photogrammetric documentation as a major additional dataset and presents it together with the flash LiDAR observations. Their joint consideration extends the published geometric evidence from sensor-focused/localized measurements to a more comprehensive representation of the submerged archaeological landscape and its erosion-related preservation context.
All pilot sites—integrated interpretation	Relevant previous TRIQUETRA publications have addressed the overall project framework, individual pilot sites, specific geometric documentation campaigns and individual sensing technologies [50,51,52,53,54,55,56,57,58,59,60,61,62,63,64,65,73].	Provides an integrated comparative interpretation of the information value and monitoring relevance of geometric documentation products across heterogeneous heritage settings. By considering all eight pilots within the same study, the analysis relates the type and scale of the generated spatial evidence to the conservation question, heritage characteristics and environmental setting, distinguishing detailed archaeological recording, documentation of terrain and cliffs, multi-temporal surface monitoring and documentation of submerged sites and underwater morphology.

**Table 2:** Comparative summary of the documentation objectives, acquisition configurations, sensor characteristics, georeferencing and validation strategies, processing software, output resolution, and monitoring-relevant indicators across the eight pilot cultural heritage sites.

	Kalapodi (Greece)	Ventotene and Santo Stefano (Italy)	Aegina (Greece)	Choirokoitia (Cyprus)
Conservation question	Stone deterioration associated with environmental weathering and freeze–thaw processes	Vulnerability of the Villa Giulia archaeological site to coastal erosion, cliff instability, landslides and rock weathering	Conservation of exposed archaeological remains in relation to coastal-cliff instability and lateral spreading of the promontory’s stiff rock cap	Site vulnerability associated with weathering, erosion, slope instability and rockfall
Intended monitoring indicator	Stone-surface deterioration/change	Cliff/slope morphological change and coastline retreat	Changes in exposed remains and adjacent cliff/slope morphology	Slope morphology and rockfall-related change through time
Survey date, acquisition geometry & spatial coverage	June 2024. Flight heights: 40 m AGL for the autonomous nadir grid and ~10–15 m AGL for manual nadir/oblique acquisition of vertical elements; site-wide sanctuary coverage	March 2023. Flight height: ~50 m. Nadir and oblique acquisition of archaeological remains and surrounding sea cliffs; ~121,562 m^2^ coverage	June 2024. Flight heights: 40 m for nadir and 15–40 m for oblique acquisition; site-wide nadir coverage, low-altitude oblique paths for walls/slopes and supplementary peripheral coverage	June 2023; May, October and November 2025. Four repeated UAV surveys; flight height: ~40 m above the highest site elevation; nadir + oblique acquisition over the archaeological site and surrounding slope
Sensor/platform & specifications	DJI Matrice 300 RTK (SZ DJI Technology Co., Ltd., Shenzhen, China); DJI P1 full-frame camera (SZ DJI Technology Co., Ltd., Shenzhen, China), 45 MP, 35 mm lens	DJI Matrice 300; DJI P1 full-frame camera, 45 MP, 35 mm lens	DJI Mavic 3 Enterprise (SZ DJI Technology Co., Ltd., Shenzhen, China); 20 MP camera, 12.29 mm focal length; DJI Mavic 2 Pro (SZ DJI Technology Co., Ltd., Shenzhen, China); 20 MP camera, 10.26 mm focal length	DJI Mavic 3 Enterprise; 20 MP camera, 12.29 mm focal length
Georeferencing/CRS/independent validation	GCPs: 13 (Leica TS10 1″, Leica Geosystems AG, Heerbrugg, Switzerland); 2 GNSS base points. Independent CPs: none. CRS: GGRS87. GCP residuals: RMSE 3D ≈ 3 mm	GCPs: 9, surveyed by GNSS. Independent CPs: none. CRS: WGS84/UTM zone 33N. GCP residuals: RMSE XY = 0.6 cm; RMSE Z = 0.93 cm; RMSE 3D = 1.1 cm	GCPs: 14. Independent CPs: 10 (Leica 1200 GNSS, Leica Geosystems AG, Heerbrugg, Switzerland; NTRIP/MetricaNET). CRS: GGRS87. GCP residuals: RMSE XY = 4.6 cm; RMSE Z = 3.0 cm; RMSE 3D = 5.5 cm. Independent CP accuracy: RMSE XY = 3.5 cm; RMSE Z = 3.3 cm; RMSE 3D = 4.8 cm	GCPs: 25. Independent CPs: 5 (Leica Viva GS10 dual-frequency GNSS, Leica Geosystems AG, Heerbrugg, Switzerland; RTK/CYPOS). CRS: CGRS93/LTM. GCP residuals: RMSE XY = 4 cm; RMSE Z = 3 cm; RMSE 3D = 5 cm. Independent CP accuracy: RMSE XY = 3 cm; RMSE Z = 8 cm; RMSE 3D = 9 cm
Processing software	RealityCapture 1.4	Agisoft Metashape Professional 2.0.1	Agisoft Metashape Professional 2.2.2	Agisoft Metashape Professional 2.2.2
Output resolution/spatial sampling	Orthomosaic GSD: 2 mm; DSM resolution: 0.5 m	Orthomosaic GSD: 1 cm; DSM resolution: 5 cm	Orthomosaic GSDs: 1 and 2.5 cm; DSM resolution: 5 cm	Orthomosaic GSD: 5 cm; DSM resolution: 10 cm
	**Smuszewo (Poland)**	**Epidaurus (Greece)**	**Roseninsel (Germany)**	**Argilliez (Switzerland)**
Conservation question	Preservation of buried wooden remains affected by water-level and moisture variability	Documentation and conservation of coastal and submerged archaeological remains	Preservation of submerged archaeological timbers in relation to lakebed conditions, sediment dynamics and water-level variation	Preservation of submerged pile-dwelling remains affected by sediment cover and erosion
Intended monitoring indicator	Water-margin variation, vegetation change and surface indicators of moisture	Changes in the geometry/visibility of submerged remains and nearshore morphology	Lakebed morphology, erosion-prone areas and water-level variation around submerged remains	Erosion-front development and sediment-cover change
Survey date, acquisition geometry & spatial coverage	September 2023–September 2025; repeated UAV surveys at 30–120 m AGL; 85% forward/60% side overlap; ~3–4 m/s; perpendicular and multi-altitude flight blocks. Coverage: ~5 ha for the fortified settlement and up to ~100 ha including the surrounding lake area	UAV photogrammetry: May 2024; autonomous wide-area nadir flights at 45 m AGL, including terrain-follow acquisition, and manual nadir/oblique detail flights at ~10–15 m AGL	Sonar: July–August 2023; wider survey of the UNESCO core and buffer zones; ~60 km tracks; 10 m spacing; 0.8 m/s; 10 Hz; ~0.32–14 m depth	Photogrammetry: January–April 2024; site-wide acquisition along 2–3 m USV corridors; ~15,000 m^2^ (~200 × 75 m)
		Underwater photogrammetry: May 2024; surface-guided dual-camera acquisition in ultra-shallow areas focused on the Roman Mansion in the south bay		
			Photogrammetry: May–July 2024; localized nadir acquisition over a high-detail area at the north-eastern tip of the island; ~1000 m^2^ at ~1 m depth	
		MBES: May 2024; bathymetric survey of the north and south bays; vessel speed ~1.5–1.8 m/s; dynamically spaced survey lines providing ~50–70% swath overlap		Flash LiDAR: repeated surveys from January to May 2025; localized USV-based survey of selected pile-dwelling remains
			Flash LiDAR: April 2025; localized survey of selected medieval bridge remains at ~7 m depth; USV speed ~0.5 m/s	
		Flash LiDAR: May 2025; localized survey of selected submerged architectural remains using a BathyDrone Sumo USV (ODAS SOLUTIONS, Annecy, France); two ~4 h raster-pattern missions with perpendicular survey directions		
Sensor/platform & specifications	Yuneec H520E (Yuneec Europe GmbH, Kaltenkirchen, Germany); E90X RGB camera (Yuneec Europe GmbH, Kaltenkirchen, Germany), 20 MP, 23 mm focal length	UAV: DJI Matrice 300 RTK; DJI Zenmuse P1 (SZ DJI Technology Co., Ltd., Shenzhen, China) full-frame RGB camera, 45 MP, 35 mm lens	Sonar: DLR La Plancha USV (German Aerospace Center, Wessling, Germany); Blue Robotics Ping single-beam echosounder (Blue Robotics Inc., Torrance, CA, USA), 115 kHz; RTK GNSS	Photogrammetry: BlueBoat USV (Blue Robotics Inc., Torrance, CA, USA); GoPro HERO10 Black (GoPro, Inc., San Mateo, CA, USA), 24 MP, 1/2.33″ sensor
		Underwater photogrammetry: Dual GoPro HERO11 Black cameras (GoPro, Inc., San Mateo, CA, USA), 27 MP, 1/1.9″ sensors, ultra-wide lenses; Hasselblad X1D II (Victor Hasselblad AB, Gothenburg, Sweden), 50 MP, 21 mm lens (15 mm full-frame equivalent)		
			Photogrammetry: LimnoVIS USV (German Aerospace Center, Wessling, Germany); Allied Vision Alvium 1900 industrial USB camera (Allied Vision Technologies GmbH, Stadtroda, Germany) equipped with a Sony IMX264 sensor (Sony Semiconductor Solutions Corporation, Atsugi, Japan), 5 MP, 6 mm lens	
		MBES: R2Sonic 2020 (R2Sonic, LLC, Austin, TX, USA); Valeport sound velocity sensor (Teledyne Valeport Ltd, Totnes, UK); Applanix SurfMaster IMU (Applanix Corporation, Richmond Hill, ON, Canada); dual Trimble GNSS receivers (Trimble Inc., Westminster, CO, USA); operating frequency: 700 kHz; swath: 90°		Flash LiDAR: CSEM Airswim direct-ToF, 532 nm; 128 × 128 array; Ekinox Micro INS; FOV: ≈ 15°; underwater measurement range: 0.7–15 m; frame rate: 4 Hz; nominal point rate: ≈65.5 kpoints/s
			Flash LiDAR: CSEM Airswim direct-ToF, 532 nm; 128 × 128 array; Ekinox Micro INS; FOV: ≈15°; underwater measurement range: 0.7–15 m; frame rate: 4 Hz; nominal point rate: ≈65.5 kpoints/s	
		Flash LiDAR: CSEM Airswim direct-ToF (CSEM SA, Neuchâtel, Switzerland), 532 nm; 128 × 128 array; Ekinox Micro INS (SBG Systems SAS, Carrières-sur-Seine, France); FOV: 18°; underwater measurement range: 0.7–15 m; frame rate: 4 Hz; nominal point rate: ≈65.5 kpoints/s		
Georeferencing/CRS/independent validation	GCPs: 6; independent CPs: typically ~6 per survey (±1–2 depending on epoch). Georeferencing: RTK camera centres via ASG-EUPOS; ground points measured by GNSS in fixed mode. CRS: PUWG 1992 (EPSG:2180); vertical datum: PL-quasi-geoid2021. GCP residuals: RMSE XY ≈ 1.2–2.5 cm; RMSE Z ≈ 2.3–3.0 cm; RMSE 3D ≈ 2.6–3.9 cm. Independent CP accuracy: RMSE XY ≈ 2–3 cm; RMSE Z ≈ 4 cm; RMSE 3D ≈ 4.5–5.0 cm	UAV photogrammetry: 24-point control network measured with dual-frequency GNSS receivers; independent CPs: none. CRS: GGRS87. GCP residual: RMSE 3D = 6.1 cm	Sonar: RTK GNSS with water-level correction; horizontal reference: ETRS89; vertical datum: DHHN2016; scale/offset depth correction based on reference measurements; 62 reference measurements using a Deeper CHIRP+ echosounder (Deeper, UAB, Vilnius, Lithuania) and tape measurements; vertical RMSE = 10 cm relative to tape measurements	Photogrammetry: 10 underwater GCPs measured using a Leica GNSS receiver (Leica Geosystems AG, Heerbrugg, Switzerland); independent CPs: none; CRS: CH1903+/LV95 (EPSG:2056); vertical reference: LN02; GCP residuals: RMSE XY ≈ 2.5–3.5 cm; RMSE Z ≈ 3.5–4.5 cm, RMSE 3D ≈ 4.5–6 cm
		Underwater photogrammetry: same 24-point control network; underwater scale bars; independent CPs: none. CRS: GGRS87. GCP residual: RMSE 3D = 5.2 cm		
			Photogrammetry: RTK GNSS + INS; 3 erosion markers used as independent references for a preliminary RTK/INS-only model (maximum deviation < 10 cm) and subsequently as GCPs in the final adjustment; no independent CPs remained for final validation. Horizontal reference: ETRS89	
		MBES: GNSS/RTK positioning with NTRIP corrections via MetricaNET and RTK-tide compensation; vertical reference/geoid model: EGM2008; positioning accuracy: ≈2 cm horizontal/5 cm vertical; independent validation: none		Flash LiDAR: INS trajectory in native GNSS coordinates; final point cloud in a local relative coordinate system referenced to the first acquisition; INS positioning accuracy: 1.4 cm horizontal/vertical; roll/pitch/heading accuracy: 0.57°; intrinsic equivalent in-water range precision ≈ 1.5 cm; repeated-line maximum deviation ≈ 3.8 cm; simultaneous LiDAR–SBES maximum depth difference ≈ 20 cm
			Flash LiDAR: INS trajectory in native GNSS coordinates; final point cloud in a local relative coordinate system referenced to the first acquisition; INS positioning accuracy: 1.4 cm horizontal/vertical; roll/pitch/heading accuracy: 0.57°; intrinsic equivalent in-water range precision ≈ 1.5 cm; independent validation: none	
		Flash LiDAR: INS trajectory in native GNSS coordinates; final point cloud in a local relative coordinate system referenced to the first acquisition; INS positioning accuracy: 1.4 cm horizontal/vertical; roll/pitch/heading accuracy: 0.57°; intrinsic equivalent in-water range precision ≈ 1.5 cm; independent validation: none		
Processing software	Pix4Dmapper 4.9	Photogrammetry: Agisoft Metashape Professional 2.1.2; MBES: Beamworx Suite 2023.2 (NAVAQ, Autoclean, TrajectEdit, AutoPatch); flash LiDAR: custom Python processing (Python 3.12.7); CloudCompare 2.13.2	Sonar: ReefMaster 2.0; photogrammetry: Agisoft Metashape Professional 2.1.1; flash LiDAR: custom Python processing (Python 3.12.7); CloudCompare 2.13.2	Photogrammetry: Agisoft Metashape Professional 2.2.2; flash LiDAR: custom Python processing (Python 3.12.7); CloudCompare 2.13.2
Output resolution/spatial sampling	Orthomosaic GSD: 1–3.5 cm; DSM/DTM resolution: ~1 cm (finest)	UAV orthomosaic GSD: 1.5 cm; underwater orthomosaic GSD: 3 mm; MBES average point density: ~700 points/m^2^; DTM sampling: 0.5 m; flash LiDAR point spacing: ~1.1 mm	Sonar raster resolution: 1 m; orthomosaic GSD: ~1 mm; flash LiDAR point spacing: ~2.4 mm	Photogrammetric orthomosaic GSD: 1.5 mm; flash LiDAR point spacing: ~1.7 mm

Abbreviations: AGL, above ground level; CP, check point; CRS, coordinate reference system; DSM, digital surface model; DTM, digital terrain model; FOV, field of view; GCP, ground control point; GNSS, global navigation satellite system; GSD, ground sampling distance; IMU, inertial measurement unit; INS, inertial navigation system; MBES, multibeam echosounder; NTRIP, Networked Transport of RTCM via Internet Protocol; RGB, red–green–blue; RMSE, root mean square error; RTK, real-time kinematic; SBES, single-beam echosounder; ToF, time of flight; UAV, unmanned aerial vehicle; USV, unmanned surface vehicle.

**Table 3:** Monitoring- and conservation-oriented interpretation of the generated products.

Documentation, Monitoring or Conservation Need	Relevant Documentation Product	Type of Information Extracted	Indicative Site Contexts
Mapping of exposed architectural remains	Orthomosaic, textured mesh, dense point cloud	Planimetric layout, wall geometry, surface continuity	Kalapodi, Aegina, Epidaurus
Interpretation of coastal or slope-related vulnerability	DSM/DTM, 3D mesh, dense point cloud	Terrain morphology, cliff/slope configuration, relation between remains and unstable landforms	Ventotene, Aegina, Choirokoitia
Recording of environmental surface change	Repeated orthomosaics and DSMs	Water-margin variation, vegetation change, surface indicators of moisture	Smuszewo
Documentation of submerged remains	Underwater photogrammetric model, flash LiDAR point cloud *	Geometry and visibility of submerged masonry or timber structures	Epidaurus, Roseninsel, Argilliez
Underwater terrain, sediment and erosion context	Bathymetric products, site-wide underwater 3D models and point clouds *	Lakebed/nearshore morphology, submerged relief, sediment context and erosion-related features	Roseninsel, Epidaurus, Argilliez
Potential spatial input to digital twin and decision support environments	Georeferenced 2D, 2.5D and 3D products	Spatial reference for linking documentation, monitoring and conservation information	All pilot sites

* For the underwater documentation categories, products listed within the same row represent operationally and informationally complementary sources of spatial evidence. They were not necessarily acquired over the same spatial extent or archaeological features, and their joint listing does not imply co-registration, fusion or quantitative inter-sensor comparison.

## Data Availability

The generated 3D geometric documentation outputs are accessible for viewing via the TRIQUETRA DSS Platform: https://dss.triquetra-project.eu/ (accessed on 27 July 2026).
